# Three New Cytotoxic *ent-*Kaurane Diterpenes from *Isodon excisoides*

**DOI:** 10.3390/molecules200917544

**Published:** 2015-09-22

**Authors:** Li-Ping Dai, Chun Li, Han-Ze Yang, Yan-Qing Lu, Hong-Yan Yu, Hui-Min Gao, Zhi-Min Wang

**Affiliations:** 1 School of pharmacy, Henan University of Traditional Chinese Medicine, Zhengzhou 450046, China; E-Mails: zzdai@163.com (L.-P.D.); yqlu123@163.com (Y.-Q.L.); 2 Institute of Chinese Materia Medica, China Academy of Chinese Medical Sciences, Beijing 100700, China; E-Mails: yujiaolong1977@aliyun.com (C.L.); huimin_gao@126.com (H.-M.G.); 3 Collaborative Innovation Center for Respiratory Disease Diagnosis and Treatment & Chinese Medicine Development of Henan Province, Zhengzhou 450046, China; 4 National Engineering Laboratory for Quality Control Technology of Chinese Herbal Medicine, Beijing 100700, China; 5 Institute of Materia Medica, Chinese Academy of Medical Sciences & Peking Union Medical College, Beijing 100500, China; E-Mail: yanghanze@imm.ac.cn; 6 Henan Province Hospital of TCM, Zhengzhou 450002, China; E-Mail: yuhongyan009@126.com

**Keywords:** *Isodon excisoides*, *ent*-kaurane diterpene, cytotoxic activity, structure-activity relationship

## Abstract

Three types of *ent*-kaurane diterpenoids were isolated from the aerial parts of *Isodon excisoides*, including three new diterpenoids, 1α,7α,14β-trihydroxy-20-acetoxy-*ent*-kaur-15-one (**1**); 1α,7α,14β,18-tetrahydroxy-20-acetoxy-*ent*-kaur-15-one (**2**); and 1α-acetoxy-14β-hydroxy-7α,20-epoxy-*ent*-kaur-16-en-15-one (**3**); together with six known diterpenes henryin (**4**); kamebanin (**5**); reniformin C (**6**); kamebacetal A (**7**); kamebacetal B (**8**); and oridonin (**9**). The structures of the isolated compounds were elucidated by means of nuclear magnetic resonance spectroscopy and high-resolution mass spectrometry in conjunction with published data for their analogs, as well as their fragmentation patterns. Compounds **5** and **9** were isolated from *Isodon excisoides* for the first time. To explore the structure-activity relationships of the isolated compounds, they were tested for their cytotoxic effects against five human cancer cell lines: HCT-116, HepG2, A2780, NCI-H1650, and BGC-823. Most of the isolated compounds showed certain cytotoxic activity against the five cancer cell lines with IC_50_ values ranging from 1.09–8.53 µM. Among the tested compounds, compound **4** exhibited the strongest cytotoxic activity in the tested cell lines, with IC_50_ values ranging from 1.31–2.07 µM. Compounds **1**, **6**, and **7** exhibited selective cytotoxic activity.

## 1. Introduction

**Figure 1:**
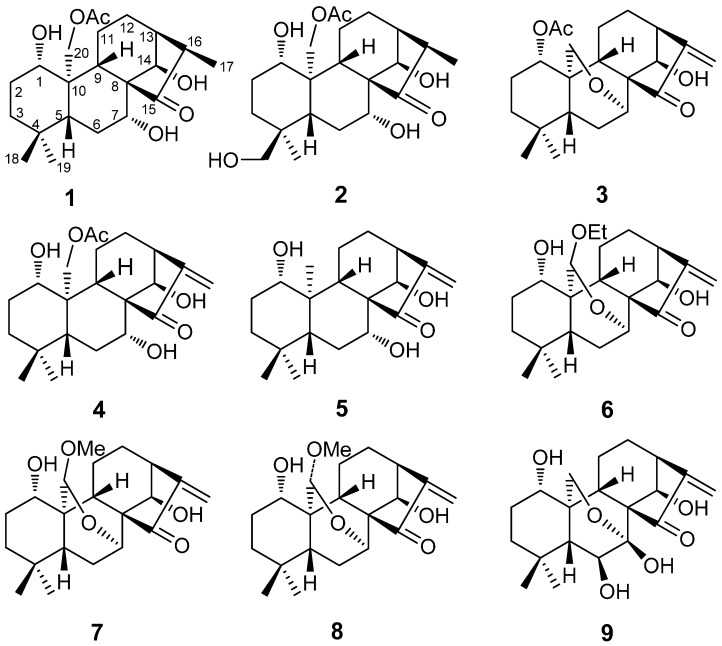
Structures of compounds **1**–**9**.

The genus *Isodon* (Lamiaceae) includes approximately 100 species of wild plants, of which 80 species and 25 varieties are found in China. About 30 species are used to treat esophageal cancer, rheumatism and chronic pharyngitis in Chinese folk medicine [1,2]. Diterpenoids are the primary bioactive constituents of *Isodon* plants. Due to their complex, fast-changing carbon skeleton and multiple pharmacological activities, diterpenoids as anti-cancer drug candidates have gained attention [3,4,5,6,7,8,9,10,11]. To date, approximately 600 diterpenoids are known to exhibit significant cytotoxicity. Cyclopentanone conjugated with an exomethylene moiety has been shown to be necessary for diterpenoid cytotoxicity [12,13]. Currently, a large number of diterpenoids have been developed into anti-cancer drugs such as oridonin, eriocalyxin A, and rabdophyllin G. Furthermore, the extracts and effective fractions enriched in diterpenoids are also approved into the market as drugs, such as *Dong-ling-cao* tablets (*Isodon rubescens*), *Xiao-yan-lidan* tablets (containing *Isodon lophanthoides* var*. graciliflorus*, as one of three ingredients), and *Weifuchun* tablets (containing *Isodon amethystoides*, as the main ingredient) [12,13]. *Isodon excisoides* (Lamiaceae) is a perennial medicinal herb widely distributed in the western region of Henan and Yunnan Provinces in China [11,14]. The aerial parts of *I. excisoides* are used to treat esophageal cancer by local people [14]. The air-dried leaves are collected and boiled in water to make tea that is consumed several times per day as a treatment for esophageal cancer. Although the chemical constituents of *Isodon* plants have been extensively studied, only approximately 20 compounds have been isolated from *I. excisoides* and its active anti-cancer constituents remain unidentified [3,15,16]. In order to search for new anti-tumor compounds and clarify the therapeutic basis of the anti-cancer effects of *I. excisoides*, we carried out a systematic phytochemical investigation on *I. excisoides*. Three new diterpenoids 1α,7α,14β-trihydroxy-20-acetoxy-*ent*-kaur-15-one (**1**); 1α,7α,14β,18-tetrahydroxy-20-acetoxy-*ent*-kaur-15-one (**2**); and 1α-acetoxy-14β-hydroxy-7α,20-epoxy-*ent*-kaur-16-en-15-one (**3**); together with six known diterpenoids, namely henryin (**4**); kamebanin (**5**); reniformin C (**6**); kamebacetal A (**7**); kamebacetal B (**8**); and oridonin (**9**), were isolated from the water extract of the aerial part of *I. excisoides.*(Figure 1). Furthermore, to explore the structure-activity relationships of the isolated compounds, the cytotoxic activities of the isolated diterpenoids were tested against five human cancer cell lines: HCT-116, A2780, NCI-H1650, BGC-823, and HepG2. Although some of the compounds isolated in our study are not new entities, to the best of our knowledge they were subjected to a consistent cytotoxic screening for the first time. In this experiment, we found that the 7,20-epoxy moiety may be associated with reduced cytotoxic activity.

## 2. Results and Discussion

### 2.1. Structure Elucidation

Compound **1** was obtained as white, needle-like crystals. The molecular formula of **1** was determined to be C_22_H_34_O_6_ on the basis of positive HR-ESI-MS at *m*/*z* 417.22367 [M + Na]^+^ (calcd for C_22_H_34_O_6_Na^+^, 417.22467). Its IR spectrum displayed the absorption bands of hydroxyl group (3422 cm^−1^) and free carbonyl group (1731 cm^−1^). The NMR spectra revealed three methyls (δ_C_ 9.2, 33.3, 21.5(each q); δ_H_ 1.12 (3H, d, 7.1 Hz), 0.91 (3H, s), 0.85 (3H, s)), one acetoxyl group (δ_C_ 170.8(s), 21.5(q); δ_H_ 2.13(3H, s)), one ketone carbonyl group (δ_C_ 222.8), three oxy-methines (δ_C_ 81.6, 76.1, 75.4) and one oxy-methylene (δ_C_ 63.7). Considering the diterpenoids previously isolated from the plant, **1** was tentatively presumed to be a 7,20-non-epoxy-*ent*-kaurane skeleton, substituted with three hydroxyl groups and one acetoxyl group.

**Figure 2:**
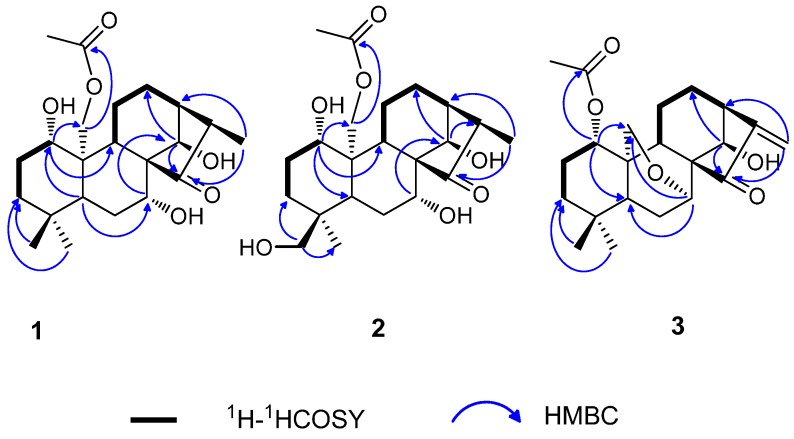
Key HMBC and ^1^H-^1^HCOSY correlations for compounds **1**–**3**.

The ^1^H- and ^13^C-NMR data of **1** were nearly identical with that of a known diterpene henryin (**4**) (Table 1) [17], and their only difference was in the moiety at C-16. For **1**, the methyl signal at δ_H_ 1.12 (3H, d, *J* = 7.2 Hz) has correlations with C-13 (δ_C_ 42.4) and C-15 (δ_C_ 222.8) in the HMBC spectrum (Figure 2), revealing that the exo-methylene at C-16 in **4** had been replaced by a methyl at C-16 in **2**.

**Table 1:** ^1^H- and ^13^C-NMR data of compounds **1**–**3** (500 and 125 MHz δ in ppm).

No.	1 (In CDCl_3_)		2 (In DMSO-*d*_6_ and D_2_O)		3 (In CDCl_3_)	
	δ_H_ (*J* in Hz)	δ_C_	δ_H_ (*J* in Hz)	δ_C_	δ_H_ (*J* in Hz)	δ_C_
1	β 3.31, dd, (11.0, 4.6)	81.6	β 3.09, dd, (10.8, 4.5)	80.5	β 4.57, dd, (11.2, 5.3)	76.6
2	α 1.82, m	30.5	α 1.63, overlapped	29.2	α 1.69, overlapped	25.1
	β 1.65, overlapped		β 1.53, overlapped		β 1.47, m	
3	α 1.47, dt, (13.8, 3.7)	39.4	α 1.53, overlapped	32.9	α 1.44, m	37.9
	β 1.29, dt, (13.8, 4.4)		β 1.05, m		β 1.24, m	
4	–	32.9	–	36.6	–	33.6
5	β 0.98, dd, (11.6, 3.5)	52.5	β 1.21, d, (12.2)	42.0	β 1.33, ddd, (11.8, 7.4, 1.5)	47.1
6	1.93, m	28.6	β 1.80, d, (12.2)	28.6	β 2.85, ddd, (14.0, 11.8, 1.9)	25.1
			α 1.63, overlapped		α 1.84, overlapped	
7	β 4.20, dd (11.4, 6.0)	75.4	β 3.77, dd, (11.8, 4.3)	73.8	β 3.95, dd, (3.8, 1.9)	64.5
8	–	61.0	–	59.8	–	58.7
9	β 1.55, d, (8.8)	55.5	β 1.37, d, (8.6)	55.7	β 1.69, overlapped	50.8
10	–	46.0	–	44.1	–	39.2
11	α 2.77, q, (6.1)	19.9	α 2.82, dd, (15.9, 5.6)	19.3	α 1.84, overlapped	17.8
	β 1.20, m		β 0.98, m		β 1.15, q, (6.5)	
12	α 1.88, m	24.2	α 1.63, overlapped	24.3	α 2.42, dt, (14.1, 9.0)	30.9
	β 1.65, overlapped		β 1.53, overlapped		β 1.54, m	
13	α 2.42, m	42.4	α 2.24, m	42.5	α 2.99, br d, (9.9)	42.0
14	α 4.82, d, (1.1)	76.1	α 4.72, br. s	75.2	α 4.61, br s	71.5
15	–	222.8	–	221.2	–	204.5
16	2.88, m	43.2	2.67, m	44.6	–	151.1
17	1.12, d, (7.1)	9.2	1.00, d, (7.1)	9.1	6.00, br s; 5.38, br.s	117.5
18	0.91, s	33.3	3.19, d, (10.6)	69.5	0.88, s	31.4
			2.84, d, (10.6)			
19	0.85, s	21.5	0.62, s	17.3	1.08, s	20.3
20	α 4.74, d, (13.2)	63.7	α 4.35, d, (13.3)	64.1	α 4.09, dd, (10.3, 1.5)	61.0
	β 4.37, d, (13.2)		β 4.28, d, (13.3)		β 4.03, dd, (10.3, 1.6)	
OAc	–	170.8	–	170.2	–	170.1
OAc	2.13, s	21.5	2.07, s	21.2	1.95, s	21.5

In the HMBC spectrum, correlations between H-1 (δ 3.31) with C-5 (δ 52.5), C-9 (δ 55.5) and C-20 (δ 63.7), H-7 (δ 4.20) with C-14 (δ_C_ 76.1), H-14 (δ 4.82) with C-12 (δ_C_ 24.2), and C-15 (δ_C_ 222.8), indicated that the hydroxy groups were at C-1, C-7, and C-14, respectively. Moreover, the acetoxyl group was assigned at C-20 based on the correlation of H-20 (δ_H_ 4.74 and 4.37) with the carbonyl at δ_C_ 170.8 (–OCOCH_3_) in the HMBC spectrum (Figure 2).

**Figure 3:**
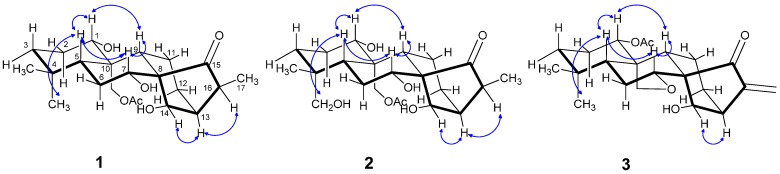
Key ROESY correlations for compounds **1**–**3**.

The relative configuration of the substituents was revealed by the ROESY spectrum, in which the correlations of H-1 with H-5 and H-9, Me-18 with H-5, H-7 with H-5 and H-9, and H-13 with H-14 and H-16, indicated that they were positioned on the same side, and that H-14, H-13, and H-16 were on the other side (Figure 3).

**Figure 4:**
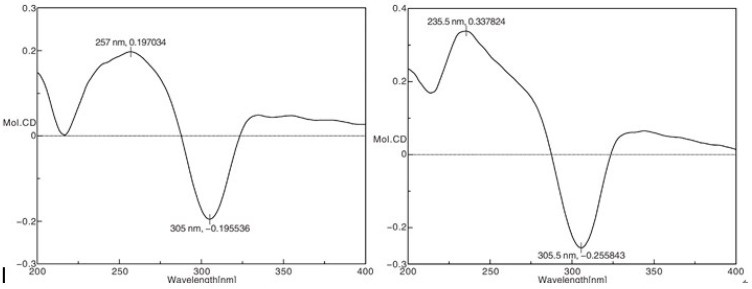
Experimental CD spectra of compounds **1** and **2**.

The absolute configuration of **1** was confirmed using the CD spectrum. According to the octant rule for saturated cyclopentanone [9], the negative Cotton effect at 305 nm (Δε-0.196), based on the n-π* transition of the saturated cyclopentanone moiety, indicated that the D ring was in a β-orientation (Figure 4). Finally, the structure of compound **1** was elucidated as 1α,7α,14β-trihydroxy-20-acetoxy-*ent*-*kaur*-15-one.

Compound **2** was obtained as a white crystal. The molecular formula was established as C_22_H_34_O_7_ by HR-ESI-MS at *m*/*z* 433.22012 [M + Na]^+^ (calcd for C_22_H_34_O_7_Na^+^, 433.21967),which indicated that **2** had an additional oxygen atom compared to **1**. The molecular formulas, NMR, and IR data suggested that **2** was an oxygenated analog of **1**. Comparison of the NMR spectral data of **2** with those of **1** indicated that one angular methyl (δ_C_ 33.3, δ_H_ 0.91 (3H, s)) at C-4 in **1** had been replaced by one hydroxymethyl (δ_C_ 69.5, δ_H_ 3.19 (1H, d, *J* = 10.6) and 2.82 (1H, d, *J* = 10.6)) in **2**. Furthermore, the downfield shift of C-4 and the upfield shift of C-3, C-5 and C-19 suggested the presence of one hydroxyl group at C-18 in **2**. The planar structure of **2** was indicated by the HMBC data (Figure 2). In the HMBC spectrum, correlations were observed for δ_H_ 3.19 (H-18) with δ_C_ 32.9 (C-3) and 17.3 (C-19) also confirmed that a hydroxymethyl group was linked to C-4.

The same relative stereo-structure for **1** and **2** was deduced from their similar ROESY correlations (Figure 3) and almost identical ^1^H- and ^13^C-NMR data. In addition, compound **2** exhibited almost the same CD absorption as that of **1** (Figure 4). Thus, the structure of **2** was determined to be 1α,7α,14β,18-tetrahydroxy-20-acetoxy-*ent*-kaur-15-one(Figure 1).

Compound **3** was obtained as a white crystalline powder. The molecular formula of **3** was deduced to be C_22_H_30_O_5_ by positive HR-ESI-MS at *m*/*z* 397.1992 [M + Na]^+^ (calcd for C_22_H_30_O_5_Na^+^, 397.19855). The UV spectrum of **3** showed an absorption maximum at 230 nm. The IR spectrum of **3** showed the presence of hydroxyl (3418 cm^−1^), carbonyl (1732 cm^−1^), and double bond (1647 cm^−1^) groups. The ^1^H- and ^13^C-NMR spectra of **3**, together with the results from a HSQC experiment showed the presence of one exocyclic double bond (δ_H_ 6.00, 5.38 (each 1H, brs); δ_C_ 117.5, 151.1), one acetoxyl group (δ_H_ 1.95 (3H, s); δ_C_ 170.1 (s), 21.5 (q)), one ketone carbonyl (δ_C_ 204.6), and two angular methyl groups (δ_H_ 0.88 and 1.08 (each 3H, s); δ_C_ 31.4 (q) and 21.3 (q)). In addition, the other carbon signals were assigned to six methenes (including one oxygenated signal), six methine carbons (including three oxygenated signals), and three quaternary carbons. These carbon signals were the characteristic signals of the structures of the diterpenoids isolated from the *Isodon genus*. The ^1^H- and ^13^C-NMR spectra of **3** were very similar to those of kamebacetal A (**7**) [18], except for the absence of a dioxygenated methine (δ_H_ 5.51 (1H, d, *J* = 1.1 Hz, H-20); δ_C_ 101.9) and a methoxyl group (δ_H_ 3.38 (3H, s); δ_C_ 54.9) as well as the presence of an acetoxyl group (δ_H_ 1.95 (s, 3H); δ_C_170.1 (s), 21.5 (q)) and an oxygenated methylene (δ_H_ 4.09 (1H, dd, *J* = 10.3, 1.5 Hz, H-20) and 4.03 (1H, dd, *J* = 10.3, 1.6 Hz, H-20); δ_C_ 61.0) in **3**. Meanwhile, the following cross-peaks were observed in the HMBC spectrum: δ_H_ 3.95 (H-7β) with δ_C_ 61.0 (C-20), δ_H_ 4.57 (H-1) with δ_C_ 170.1 (OAc), and δ_H_ 4.61 (H-14) with δ_C_ 30.9 (C-12), 204.5 (C-15) and 151.1 (C-16). Thus, the basic skeleton of **3** was assumed to be 1-acetoxy-14-hydroxy-7,20-epoxy-*ent*-kaur-16-en-15-one.

The relative configuration of **3** was revealed by ROESY experiments (Figure 3). In the ROESY spectrum, the correlations of H-1/H-5 and H-9, Me-18/H-5, H-7/H-5 and H-9, H-14/H-13 were observed. These results indicated that H-1, H-5, H-9, and Me-18 should be on the same side of the molecule, and H-14 and H-13 should be on the other side.

The negative Cotton effect at 356 nm (Δε-0.02), based on the n-π* transition of the unsaturated cyclopentanone moiety, indicated that the D ring was in a β-orientation [11]. According to the analysis described above, the structure of **3** was determined to be 1α-acetoxy-14β-hydroxy-7α,20-epoxy-*ent*-kaur-16-en-15-one.The six known compounds isolated from *I.excisoides* were identified as henryin (**4**) [17], kamebanin (**5**) [19], reniformin C (**6**) [20], kamebacetal A (**7**) [18], kamebacetal B (**8**) [21], and oridonin (**9**) [22], by comparison of their spectral data to the reported in the literature.

### 2.2. Cytotoxicity Assay

Using the MTT method [23], all compounds were evaluated for their cytotoxic effects against five human cancer cell lines HCT-116, A2780, NCI-H1650, BGC-823, and HepG2. Most of the tested compounds exhibited potent cytotoxicity. The results are presented in Table 2.

**Table 2:** Cytotoxic activities of all tested compounds on five human cancer cell lines.

Sample	IC_50_ (μM)				
	HCT-116	HepG2	BGC-823	NCI-H1650	A2780
1	2.94 ± 0.06	3.07 ± 0.02	5.59 ± 0.19	>10	6.33 ± 0.34
2	2.45 ± 0.12	3.21 ± 0.09	4.17 ± 0.25	>10	5.61 ± 0.19
3	2.13 ± 0.81	2.20 ± 1.12	>10	5.68 ± 0.73	1.09 ± 0.13
4	1.77 ± 0.22	1.54 ± 0.32	1.31 ± 0.76	2.07 ± 0.36	1.42 ± 0.20
5	4.85 ± 0.33	7.88 ± 1.02	2.99 ± 0.76	>10	1.56 ± 0.34
6	4.81 ± 0.01	7.45 ± 0.22	5.17 ± 0.61	>10	1.98 ± 0.13
7	4.87 ± 1.12	1.09 ± 0.06	5.31 ± 0.14	2.58 ± 0.23	1.44 ± 0.07
8	4.55 ± 0.02	7.35 ± 0.48	4.97 ± 0.84	2.38 ± 0.31	4.88 ± 0.22
9	4.85 ± 0.33	7.88 ± 1.02	2.99 ± 0.76	>10	1.56 ± 0.34
DDP	7.81 ± 0.14	>10	8.56 ± 1.05	>10	8.65 ± 0.59
Taxol	(3.07 ± 0.12) × 10^−2^	(1.31 ± 0.44) × 10^−2^	(4.06 ± 0.35) × 10^−3^	(2.61 ± 1.02) × 10^−2^	(7.13 ± 0.51) × 10^−3^

DDPH (cisplatin) and taxol were used as positive controls.

### 2.3. Analysis of Structure-Activity Relationships

We assessed the structure-activity relationships of the isolated compounds, based on the results of cytotoxic activity test. Compounds **1**, **2**, **4**, and **5** were 7, 20-non-epoxy kaurane diterpenoids and were present in large amounts in *I. excisoides*. Compounds **4** and **5** contained α,β-unsaturated pentone and exocyclic methylene. In addition, compound **4** also contained 20-OAc. No exocyclic double bond was found in compounds **1** or **2**; however, 20-OAc was present in these compounds. Previous reports have suggested that α,β-unsaturated pentones and exocyclic methylene are essential structural requirements for the cytotoxic activity of diterpenoids [24,25,26]. The results of the cytotoxic activity tests indicated that all 4 compounds had cytotoxic activity. Although the NCI-H1650 cell line was resistant to compounds **1** and **2**, these compounds exhibited significant cytotoxic activities (IC_50_: 2.94–6.47 μM) against the other four tumor cell lines, while compound **4** displayed the highest cytotoxic activity. This suggests that α,β-unsaturated pentone and exocyclic methylene are not the only moieties required for the cytotoxic activity of diterpenoids. Furthermore, 7,20-non-epoxy kaurane diterpenoid, and 20-OAc may also be responsible for the cytotoxic activity of diterpenoids. this possibility should be further investigated.

Compounds **3**, **6**, **7**, **8**, and **9** are 7,20-epoxy kaurane diterpenoids composed of α,β-unsaturated pentone and exocyclic methylene. A large number of studies have confirmed that compound **9** has definite cytotoxic activity [25,26,27]. Pre-clinical studies are being conducted on the use of compound **9** as a potential new drug. Compounds **3** and **6**–**9** exhibited expected levels of cytotoxic activity consistent with previous reports suggesting that α,β-unsaturated pentones and exocyclic methylene are responsible for the cytotoxic activity of diterpenoids.

Compounds **6**–**8** are variants of diterpenoids comprising an oxygen-containing substituent on the C-20 chiral carbon, but have different configurations (compounds **6** and **7** have an α-configuration, while compound **8** has a β-configuration). A desirable result is that compounds of different configurations display selective activity against NCI-H1650 and HepG2 cell lines. Compound **8** exhibited significant cytotoxic effects on both NCI-H1650 and HepG2 cells (IC_50_: 1.09–2.58 μM), whereas compounds **6** and **7** had an insignificant effect on the NCI-H1650 cell line (IC_50_ > 10 μM), suggesting that the relative configuration of the C-20 chiral carbon affected the cytotoxic effect of the tested diterpenoids on some of the tumor cell lines (NCI-H1650 and HepG2), whereas the β-configuration enhanced their cytotoxic activity.

Among the tested compounds, compound **4** exhibited the highest cytotoxic activity against the five tested tumor cell lines (IC_50_: 1.31–2.07 μM). The cytotoxic activity of compound **4** was almost 5**-**fold higher than that of compound **9**. These results indicate that 7,20-non-epoxy, α,β-unsaturated pentones and exocyclic methylene, as well as the 20-OAc group, had a positive effect on the cytotoxic activity of diterpenoids. α,β-unsaturated pentones (compounds **1** and **2**), exocyclic methylene, and configurations of C-20 chiral carbon (compounds **6**–**8**) significantly affected the cytotoxic activity of the tested diterpenoids in some of the cell lines, such as NCI-H1650 and HepG2.

## 3. Experimental Section

### 3.1. General Information

Melting points were measured on a Boetius micro melting point apparatus and were uncorrected. IR spectra were recorded on a Spectrum 100 FT-IR Spectrometer (PerkinElmer, Waltham, MA, USA). UV spectra were recorded on a Shimadzu double-beam 210A spectrophotometer (Shimadzu, Kyoto, Japan). Optical rotation was measured using a SEPA-300 polarimeter (Horiba, Tokyo, Japan). NMR spectra were recorded on a Bruker Avance III spectrometer (Bruker, Billerica, Germany) with TMS as the internal standard. Chemical shifts (δ) are expressed in ppm with reference to the solvent signals. HRESIMS data was acquired using an LTQ orbitrap (Thermo Fisher Scientific, Inc., Bremen, Germany). Semi-preparative HPLC was performed on a Waters 600/Waters 2487 (Waters, Milford, MA, USA) with a YMC (250 mm × 10 mm I.D. 5 μm) column. Column chromatography was performed either on silica gel (100–200 mesh and 200–300 mesh, Qingdao Marine Chemical Inc., Qingdao, China) or on MCI gel CHP 20P (75–150 μm, Mitsubishi Chemical Corp., Tokyo, Japan), ODS (50 μm, YMC, Kyoto, Japan), Sephadex LH-20 (Pharmacia Biotech AB, Uppsala, Sweden). Solvents were distilled prior to use. Spectroscopic grade solvents were used. TLC was carried out on pre-coated silica gel HF_254_ plates. Spots were visualized by heating silica gel plates sprayed with 20% H_2_SO_4_ in ethanol (*v/v*).

### 3.2. Plant Material

The aerial parts of *I. excisoides* were collected from Luanchuan County in Henan Province, China, in September 2011 and authenticated by professor Xiao-Zheng Luo of the Henan College of Traditional Chinese Medicine. A voucher specimen (No. 2011-0905) was deposited in the Key Laboratory of Traditional Chinese Medicine Chemistry and Resource of Henan Province.

### 3.3. Extraction and Isolation

The air-dried and powdered aerial parts of *I. excisoides* (10 kg) were decocted three times with water (320 L × 1.5 h) at 100 °C. The decoction was then evaporated to obtain a concentrate with a concentration equivalent to 0.1 g/mL of crude drug. The concentrate was subjected to chromatography on D-101 macroporous resin and successively eluted with different concentrations of EtOH (0, 30%, 70% and 95% EtOH), to obtain four fractions (Fr. A–D).

Fr. B (20 g, 30% EtOH elute) was subjected to column chromatography over MCI gel (50 cm× 4 cm) and eluted with MeOH–H_2_O (30:70, 1.5 L; 50:50, 2.5 L; 100:0, 1 L) to yield three subfractions (Fr. B_1_–B_3_). Compound **4** (40 mg) was crystallized from Fr. B_2_ using MeOH. Fr. B_1_ (5 g) was further subjected to column chromatography on a Sephadex LH-20 column and eluted with MeOH–H_2_O (30:70) to afford a mixture of three diterpenoids, which was further purified by semi-preparative HPLC (MeOH–H_2_O, 33:67, 2.7 mL/min). Detection was monitored at 190, 190, and 230 nm. Compounds **1** (8 mg), **2** (7 mg) and **9** (10 mg) were obtained at 28.4, 26.9, and 21.0 min, respectively. Fr. C (36 g, 70% EtOH elute) was further chromatographed on MCI gel (50 cm× 4 cm), eluted with MeOH–H_2_O (30:70 1.5 L; 50:50 3.5 L; 100:0 1 L) to yield three subfractions (Fr. C_1_–C_3_). Compound **7** (70 mg) was crystallized from Fr. C_2_ using CHCl_3_. The Fr. C_2_ residue was subjected to column chromatography over silica gel (100–200 mesh), eluted with petroleum ether–Me_2_CO (10:1, 8:1, 5:1, 3:1, 0:1) to afford compounds **5** (15 mg) and **8** (15 mg). Compounds **3** (8 mg) and **6** (4 mg) were purified after repeated chromatography over silica gel and a RP-18 column (30%–100% MeOH) from the petroleum ether–Me_2_CO (5:1) fraction of Fr. C_2_.

*1α,7α,14β-Trihydroxy-20-acetoxy-ent-kaur-15-one* (**1**): white crystals (MeOH); mp: 235 °C; ${\left[\alpha \right]}_{D}^{20}$ −27.02 (*c* 0.18, MeOH), UV (MeOH) λ_max_ (log ε): no absorption; IR (KBr) λ_max_ (cm^−1^): 3544, 3422, 2931, 2875, 1731, 1465, 1381, 1253, 1093, 1062, 1043 cm^−1^; HRESIMS *m*/*z*: 417.22367 [M + Na]^+^ (calcd for C_22_H_34_O_6_Na^+^, 417.22467); CD (MeOH) λ_max_ (Δε) 257 (+0.20), 305 (−0.20) nm; See Table 1 for ^1^H-NMR (CDCl_3_, 500 MHz) and ^13^C-NMR (CDCl_3_, 125 MHz) spectral data.

*1α,7α,14β,18-Tetrahydroxy-20-acetoxy-ent-kaur-15-one* (**2**): white crystals (MeOH); mp: 231 °C; ${\left[\alpha \right]}_{D}^{20}$ −19.48 (*c* 0.77, MeOH); UV (MeOH) λ_max_ (log ε):no absorption; IR (KBr) λ_max_ (cm^−1^): 3473, 2918, 2866, 1737, 1725, 1485, 1377, 1269, 1082, 1056, 957 cm^−1^; HR-ESI-MS *m*/*z*: 433.22012 [M + Na]^+^ (calcd for C_22_H_34_O_7_Na^+^, 433.21967); CD (MeOH) λ_max_ (Δε) 236 ( +0.34), 306 (−0.26) nm; See Table 1 for ^1^H-NMR (DMSO-*d*_6_ + D_2_O, 500 MHz) and ^13^C-NMR(DMSO-*d*_6_ + D_2_O, 125 MHz) spectral data.

*1α-Acetoxy-14β-hydroxy-7α,20-epoxy-ent-kaur-16-en-15-one* (**3**): white crystalline powder; mp:165 °C; ${\left[\alpha \right]}_{D}^{20}$ −50 (*c* 0.06, MeOH); UV (MeOH) λ_max_ (log ε): 230 (3.52) nm; IR (KBr) λ_max_ (cm^−1^): 3418, 2959, 2871, 1732, 1647, 1496, 1372, 1242, 1078, 1030, 984 cm^−1^; HRESIMS *m*/*z*: 397.19923 [M + Na]^+^ (calcd for C_22_H_30_O_5_Na^+^, 397.19855); CD (MeOH) λ_max_ (Δε) 223 (+3.43), 284 (+1.19), 356 (−0.02) nm; See Table 1 for ^1^H-NMR (CDCl_3_, 500 MHz) and ^13^C-NMR (CDCl_3_, 125 MHz) spectral data.

### 3.4. Cytotoxicity Assay

Five human cancer cell lines (colon carcinoma cell line HCT-116, hepatic cancer cell line HepG2, ovarian cancer cell line A2780, lung cancer cell line NCI-H1650, and gastric cancer cell line BGC-823) were used. All cells were cultured in RPMI-1640 medium, supplemented with 10% fetal bovine serum in a humidified atmosphere with 5% CO_2_ at 37 °C. The cytotoxicity assay was performed according to the MTT (3-(4,5-dimethylthiazol-2-yl)-2,5-diphenyl tetrazolium bromide) method using 96-well microplates [28]. Briefly, the cells were cultured in RPMI-1640 medium supplemented with 10% fetal bovine serum in a humidified atmosphere with 5% CO_2_ at 37 °C. Next, 100 μL of adherent cells at a density of 5 × 10^4^ cell/mL were seeded into each well of the 96-well cell culture plates and incubated in 5% CO_2_ at 37 °C for 24 h to form a monolayer on the flat bottom. Next, the supernatant of each well was removed, after which 100 μL of fresh medium and 100 μL of medium containing a test sample were added to the well. The plate was then incubated in 5% CO_2_ at 37 °C for 72 h. Next, 20 μL of 5 mg/mL MTT in DMSO was added to each well and further incubated for 4 h. The supernatant was carefully removed from each well and 150 μL of DMSO were added. The plate was then vortex-shaken for 15 min to dissolve the blue formazan crystals. The optical density (OD) of each well was measured on a Genois microplate reader (Tecan GENios, Männedorf, Switzerland) at a wavelength of 570 nm.

In each experiment, each tumor cell line was exposed to the test compound at concentrations of 1 × 10^−5^, 1 × 10^−6^, and 1 × 10^−7^ mol/L. The inhibitory rate of the cell growth was calculated according to the following formula: Inhibition rate (%) = (OD_control_ − OD_treated_)/OD_control_ × 100. Finally, IC_50_ values were calculated using SPSS 16.0 statistical software.

## 4. Conclusions

Phytochemical investigations on the water extract of the aerial parts of *I. excisoides* resulted in the isolation of three new compounds **1**–**3**, along with six known compounds **4**–**9**. The isolated compounds were identified as 1α,7α,14β-trihydroxy-20-acetoxy-*ent*-kaur-15-one (**1**); 1α,7α,14β,18-tetrahydroxy-20-acetoxy-*ent*-kaur-15-one (**2**); 1α-acetoxy-14β-hydroxy-7,20-epoxy-*ent*-kaur-16-en-15-one (**3**); henryin (**4**); kamebanin (**5**); reniformin C (**6**); kamebacetal-A (**7**); kamebacetal B (**8**); and oridonin (**9**). Compounds **5** and **9** were isolated from *I. excisoides* for the first time.

All the compounds were evaluated for their cytotoxic effects against five human tumor cell lines (HCT-116, HepG2, A2780, NCI-H1650, and BGC-823) and all showed certain cytotoxic activity against all five types of human tumor cells. Furthermore, we studied the cytotoxic activity of kaurane diterpenoids with different structural features for the first time. It was observed that diterpenoids with a 7,20-non-epoxy group, α,β-unsaturated pentones and an exocyclic methylene group, the 20-OAc group can improve cytotoxic activity. These results suggest that the cyclopentanone conjugated with an exomethylene group is mainly responsible, although not the sole factor, for the cytotoxic activity of 7,20-non-epoxy diterpenoids. Diterpenoids containing the 20-OAc group also displayed significant cytotoxic activity (compounds **1** and **2**). The presence of the 20-OAc group has a positive effect on the cytotoxic activity (compounds **1**, **2**, and **4**), and the mechanism underlying their cytotoxic activity requires further investigation. There have been many reports on 7, 20-epoxy kaurane diterpenoids [23,24,25,26,27]. Our study revealed that the configuration of the C-20 chiral carbon had a positive effect on the cytotoxic activity. In addition, β-configuration of C-20 (compound **8**) showed marked selective cytotoxic activity against the NCI-H1650 and HepG2 cell lines. More similar compounds should be used to further study this structure-activity relationship in the future.

## Supplementary Materials

The IR, HR-ESI-MS, NMR (1D and 2D) and CD spectra of compounds **1–3** are available as supporting information. Supplementary materials can be accessed at: http://www.mdpi.com/1420-3049/20/09/17544/s1.
