# Supplementary material for: Three New Cytotoxic ent-Kaurane Diterpenes from Isodon excisoides
Source: Molecules. 2015 Sep 22;20(9):17544–56. doi: 10.3390/molecules200917544 (PMC6332174; doi:10.3390/molecules200917544)
Supplement: Supplementary file 1 [file molecules-20-17544-s001.pdf]

Supplementary File

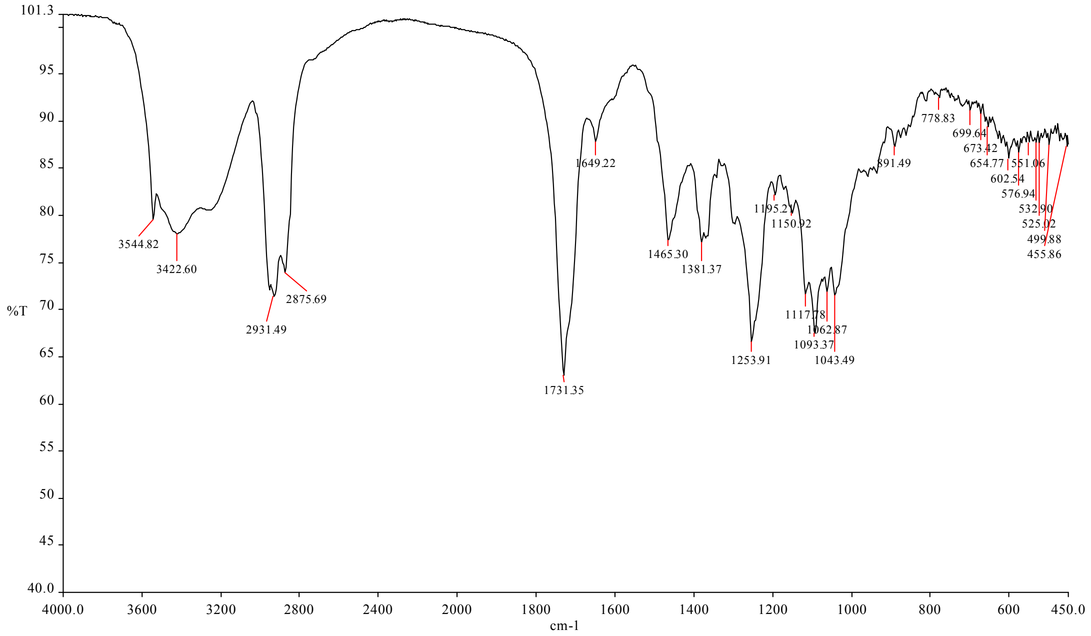

Figure S1. IR spectrum of compound **1**.

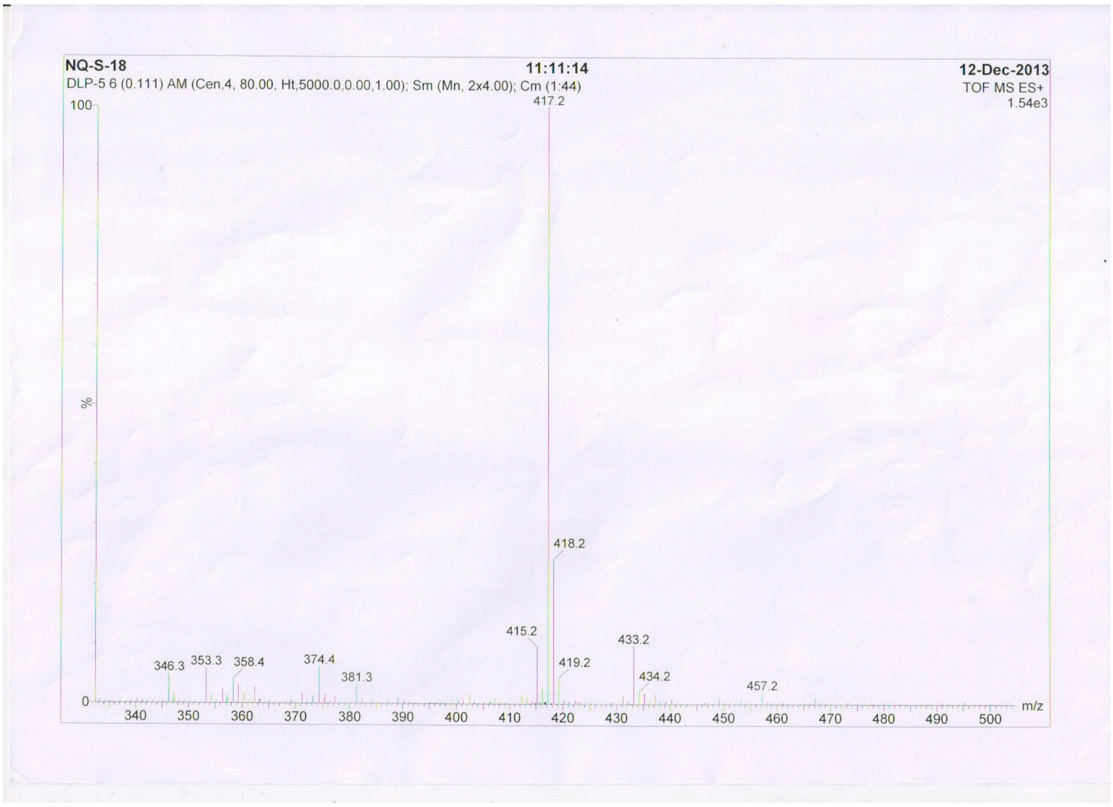

Figure S2. ESI-MS spectrum of compound **1**.

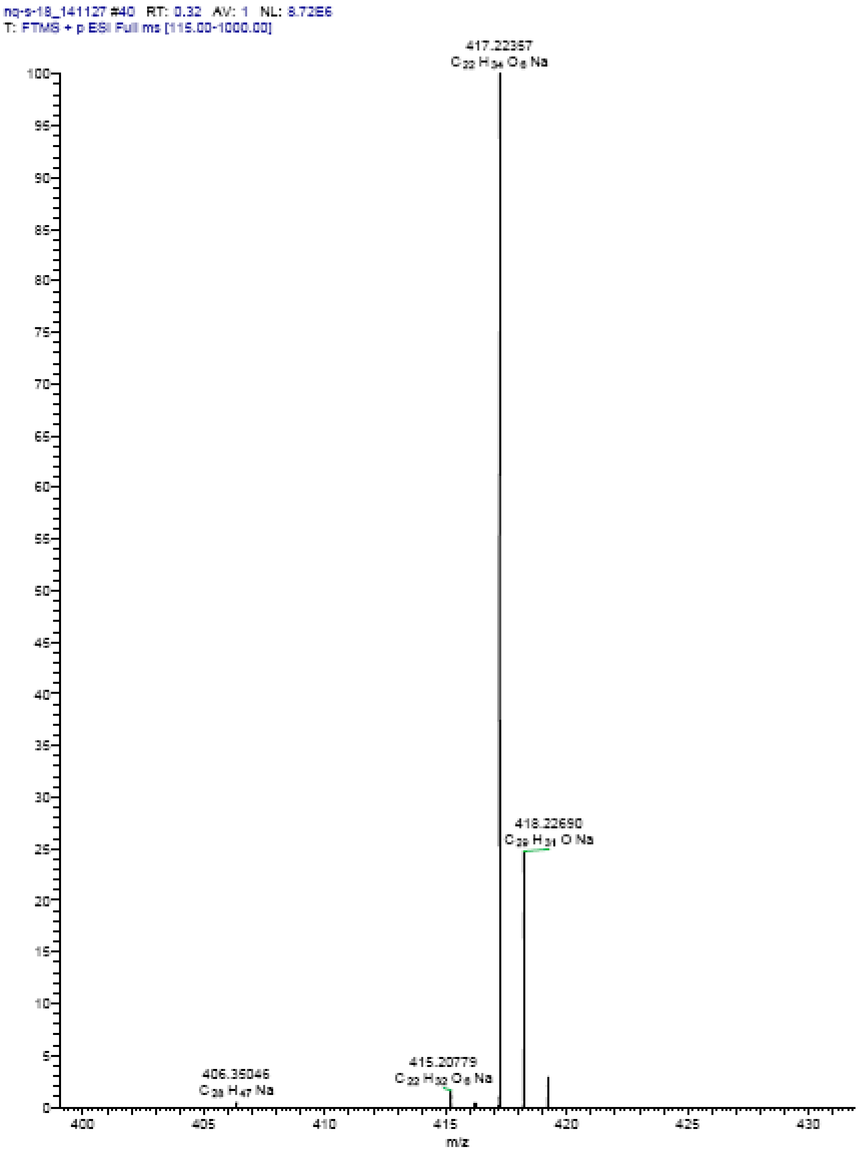

Figure S3. HR-ESI-MS spectrum of compound **1**.

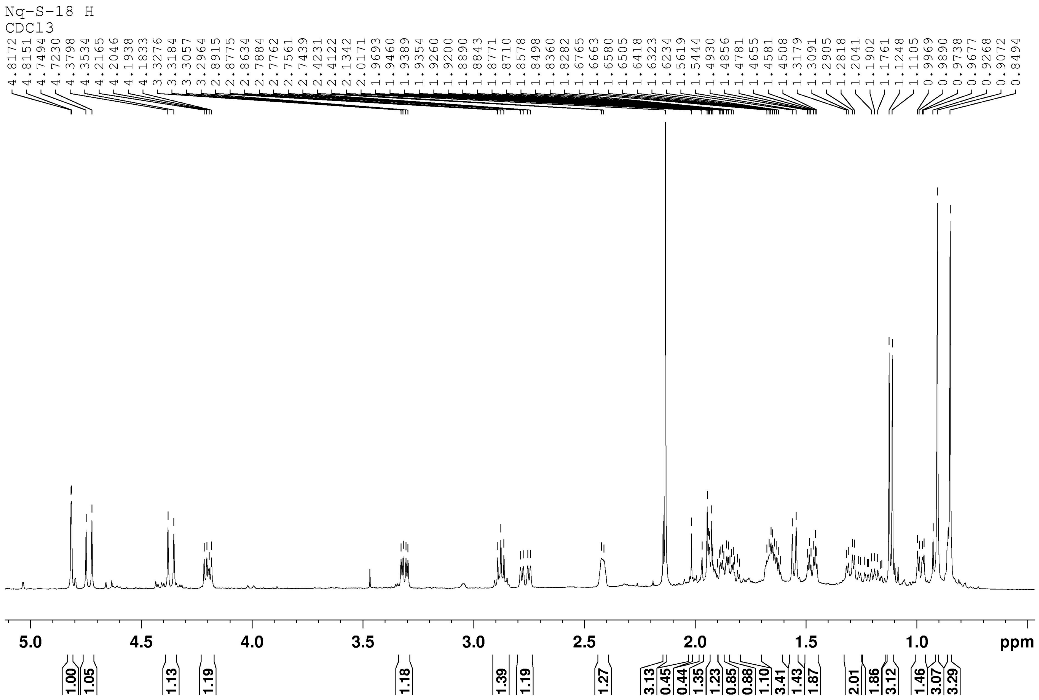

Figure S4. ^{1}H-NMR spectrum of compound **1**.

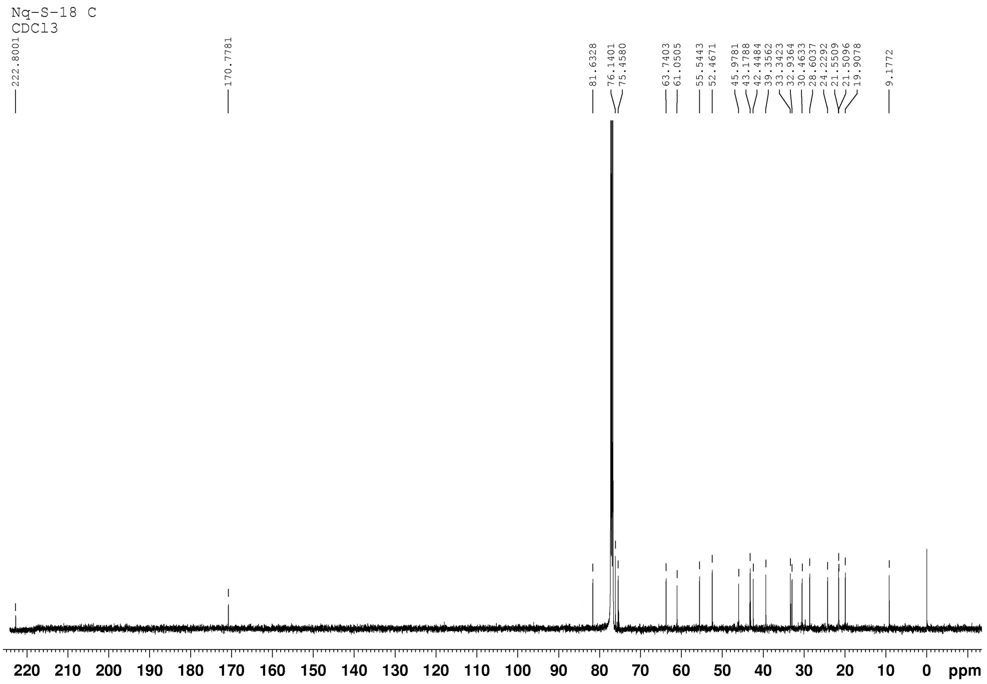

Figure S5. ^{13}C-NMR spectrum of compound **1**.

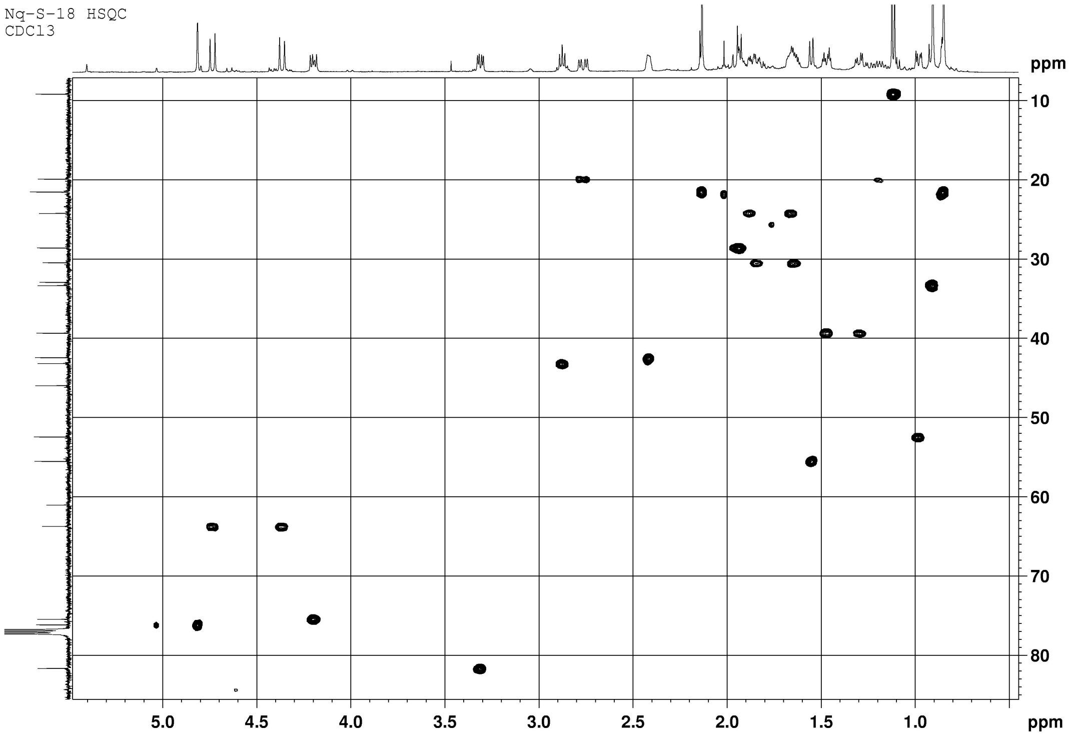

Figure S6. HSQC spectrum of compound **1**.

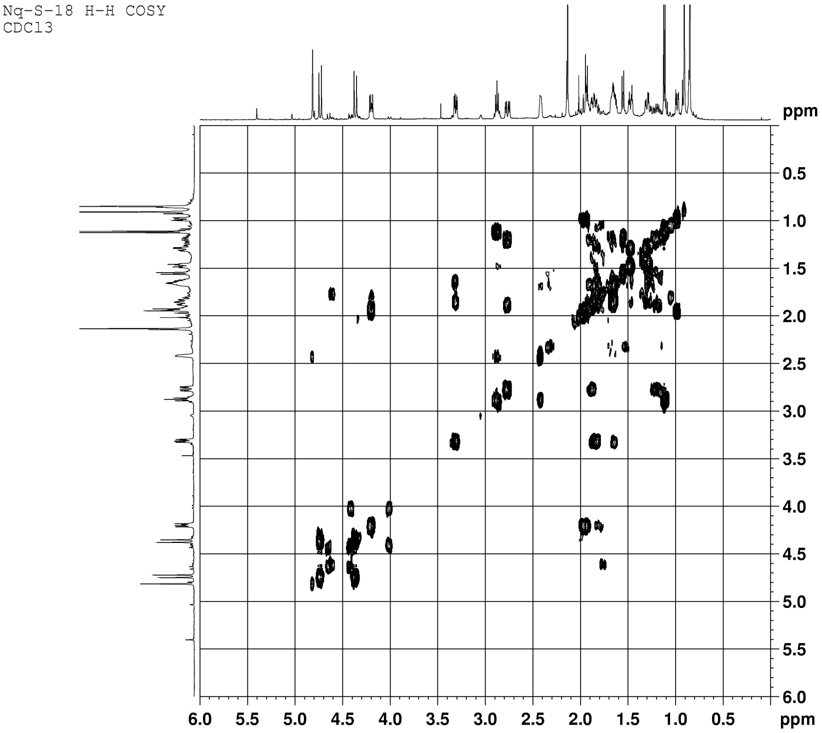

Figure S7. ^{1}H- ^{1}H COSY spectrum of compound **1**.

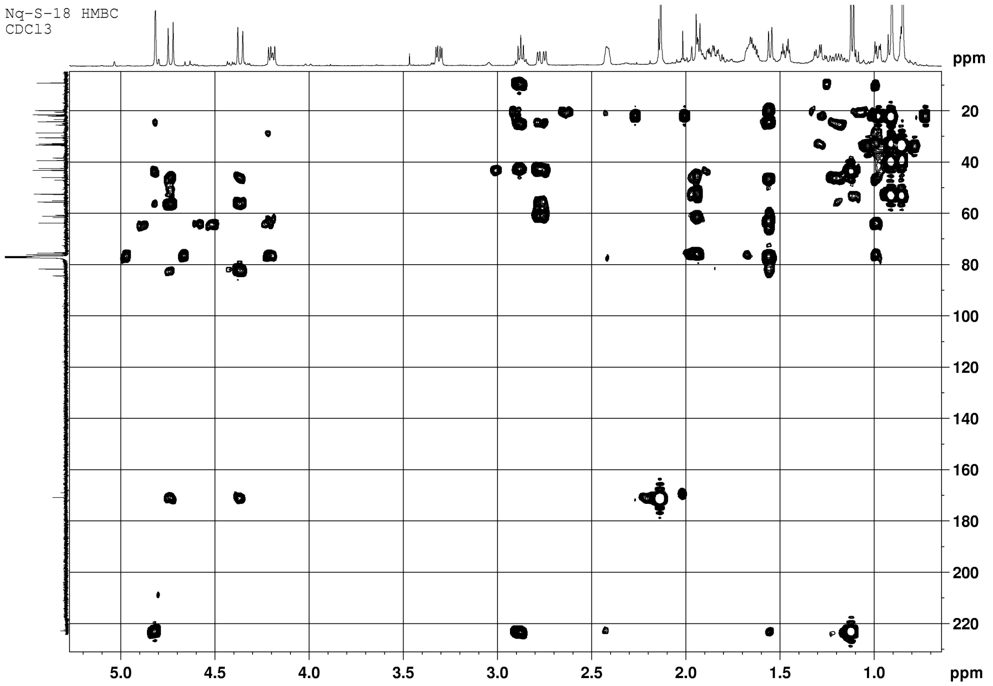

Figure S8. HMBC spectrum of compound **1**.

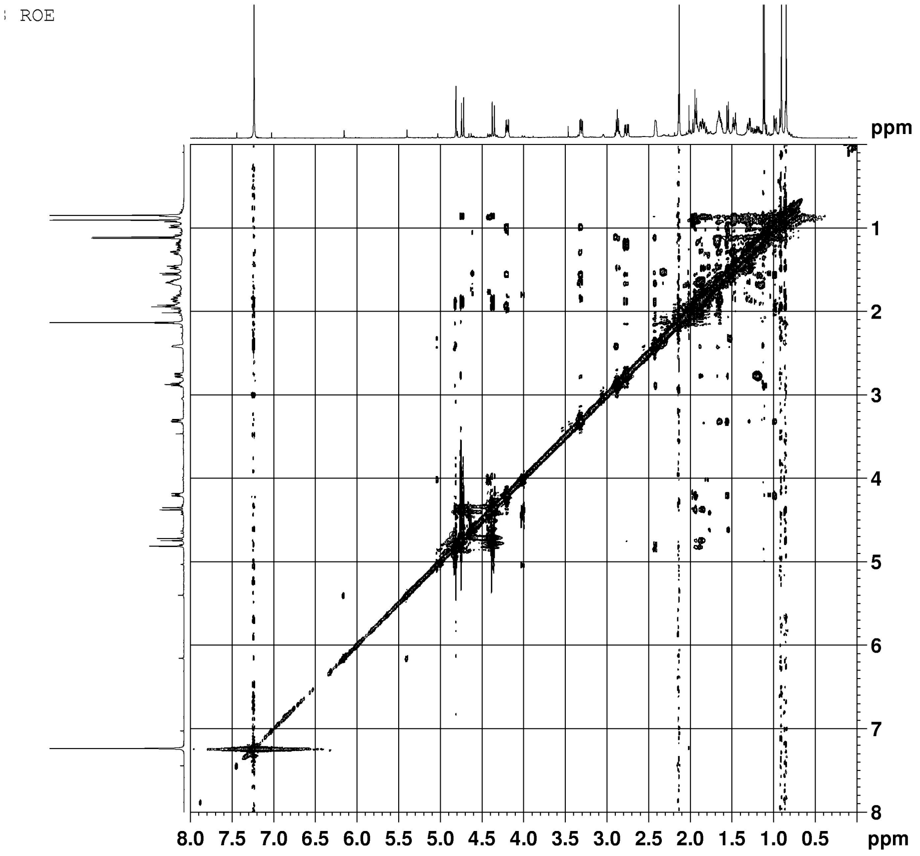

Figure S9. ROESY spectrum of compound **1**.

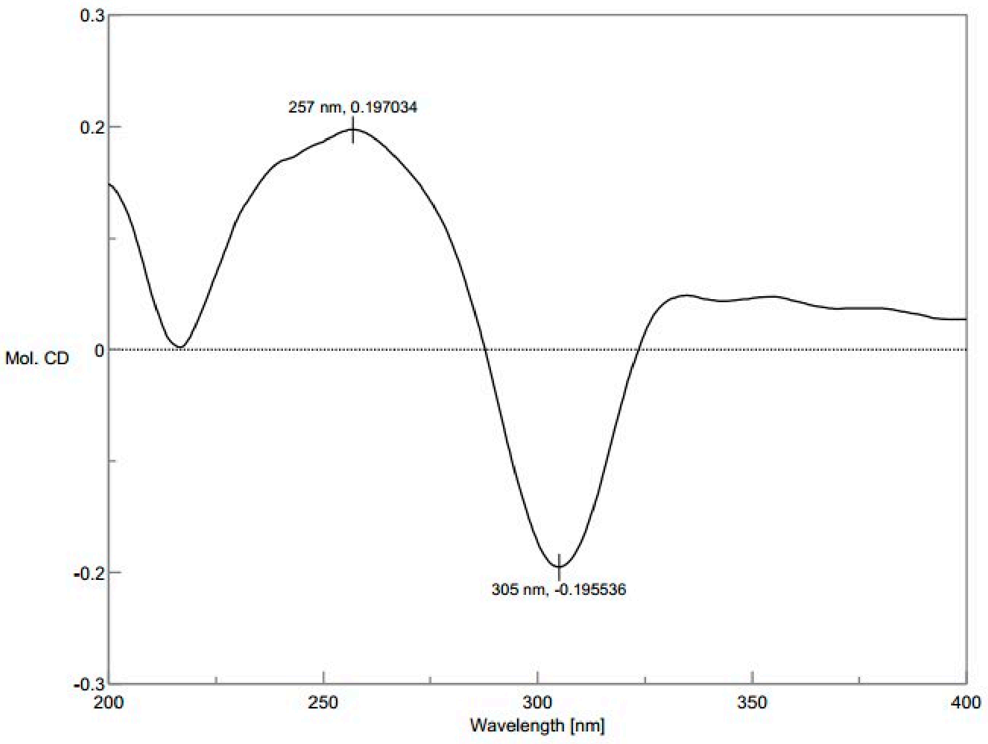

Figure S10. CD spectrum of compound **1**.

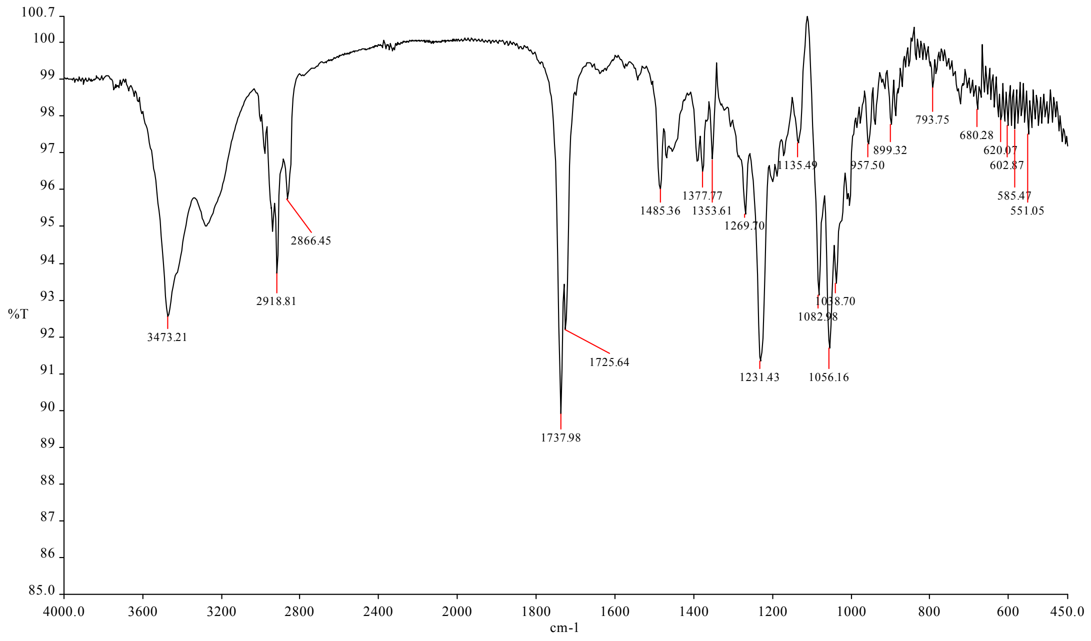

Figure S11. IR spectrum of compound **2**.

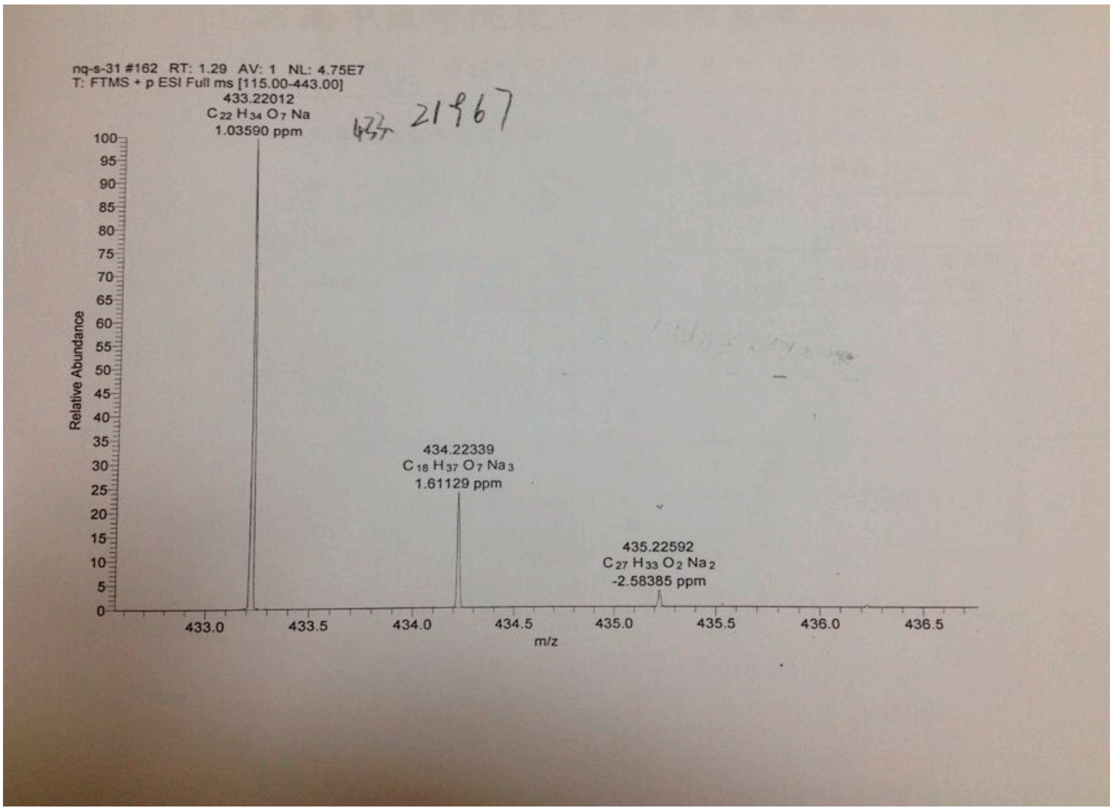

Figure S12. HR-ESI-MS spectrum of compound **2**.

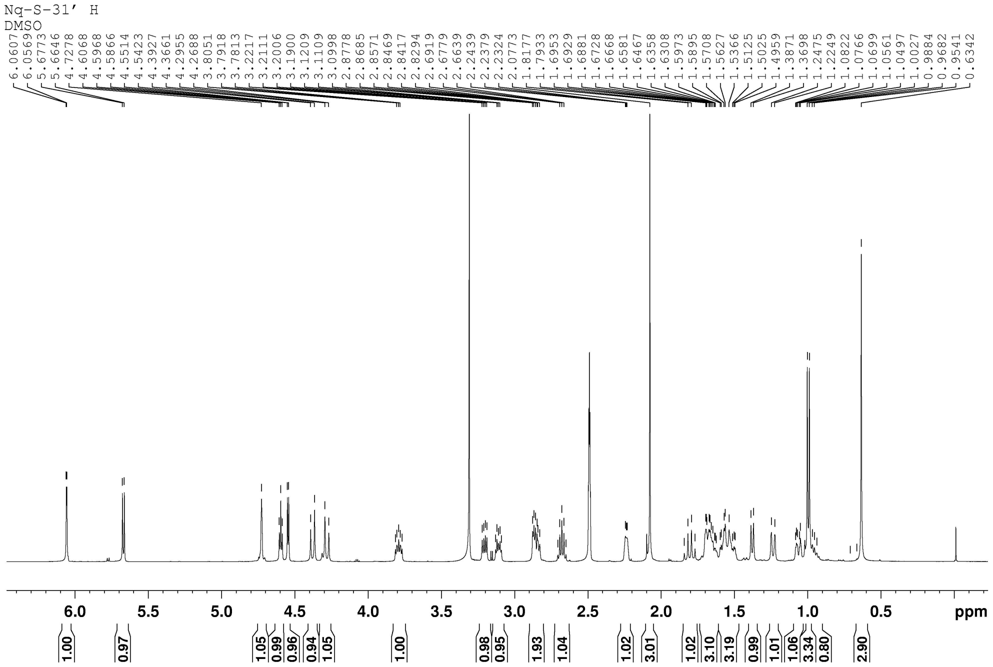

Figure S13. ^{1}H-NMR spectrum of compound **2**.

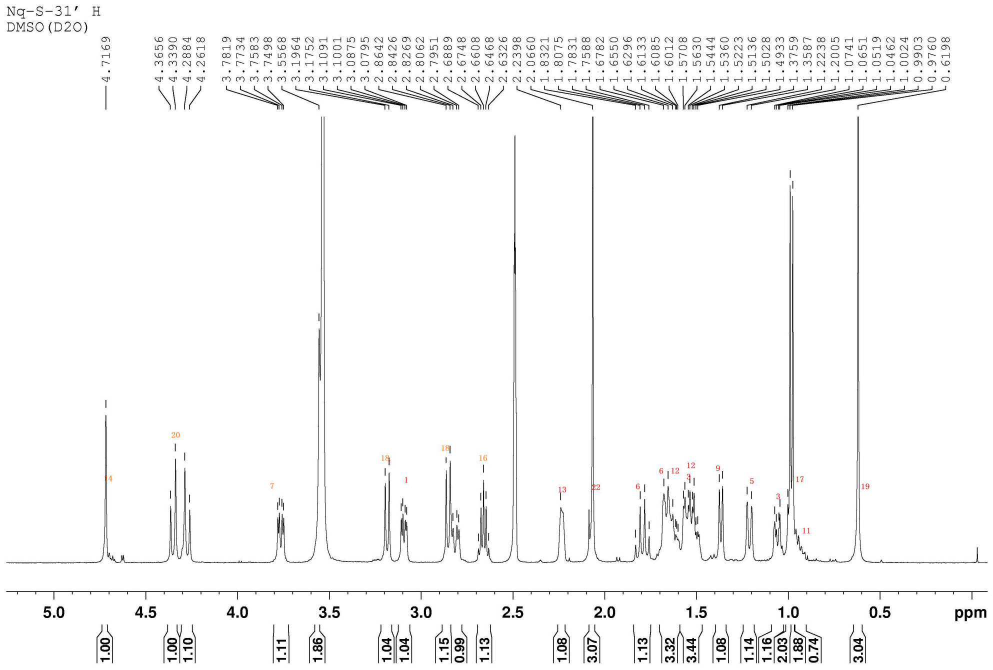

Figure S4. ^{1}H-NMR spectrum of compound **2**.

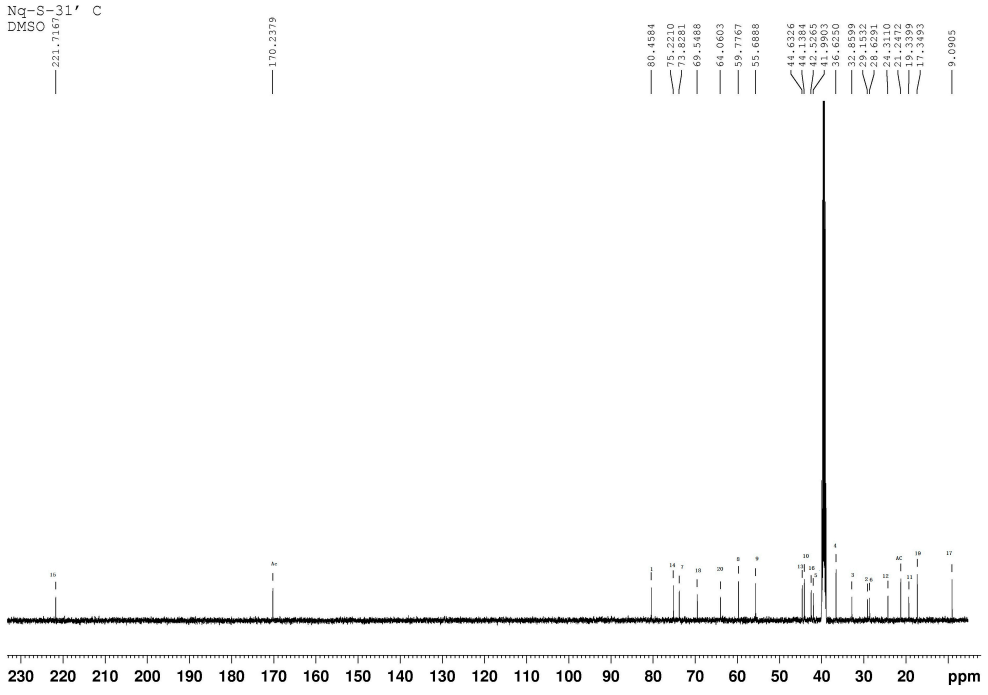

Figure S15. ^{13}C-NMR spectrum of compound **2**.

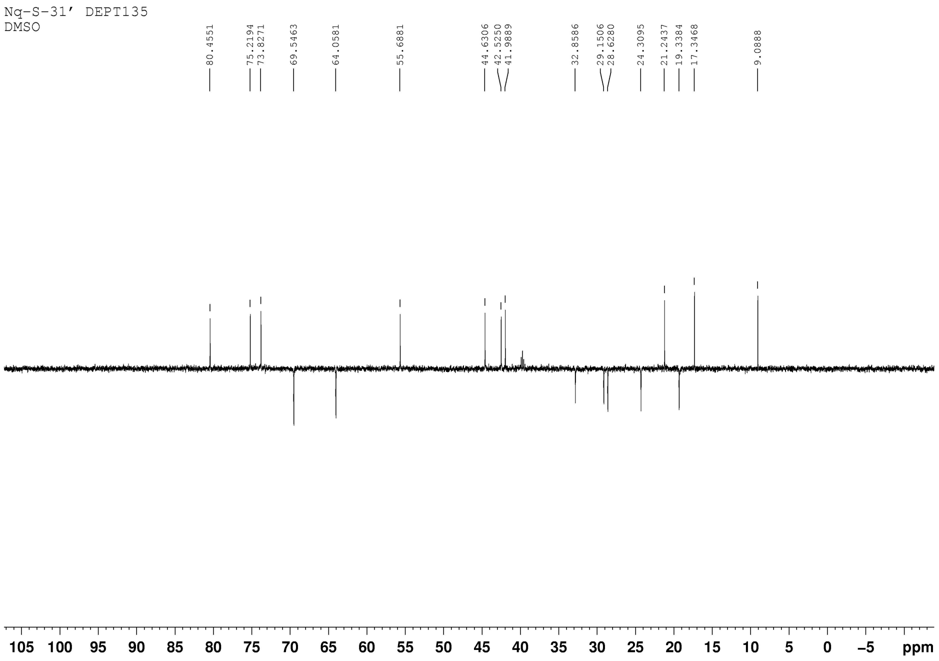

Figure S16. DEPT spectrum of compound **2**.

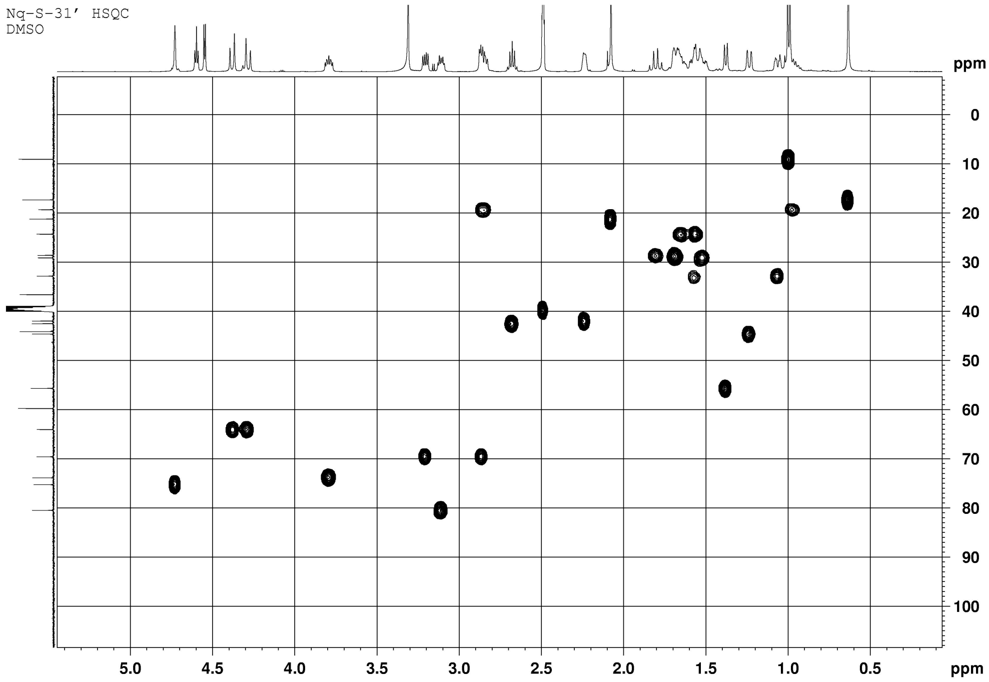

Figure S17. HSQC spectrum of compound **2**.

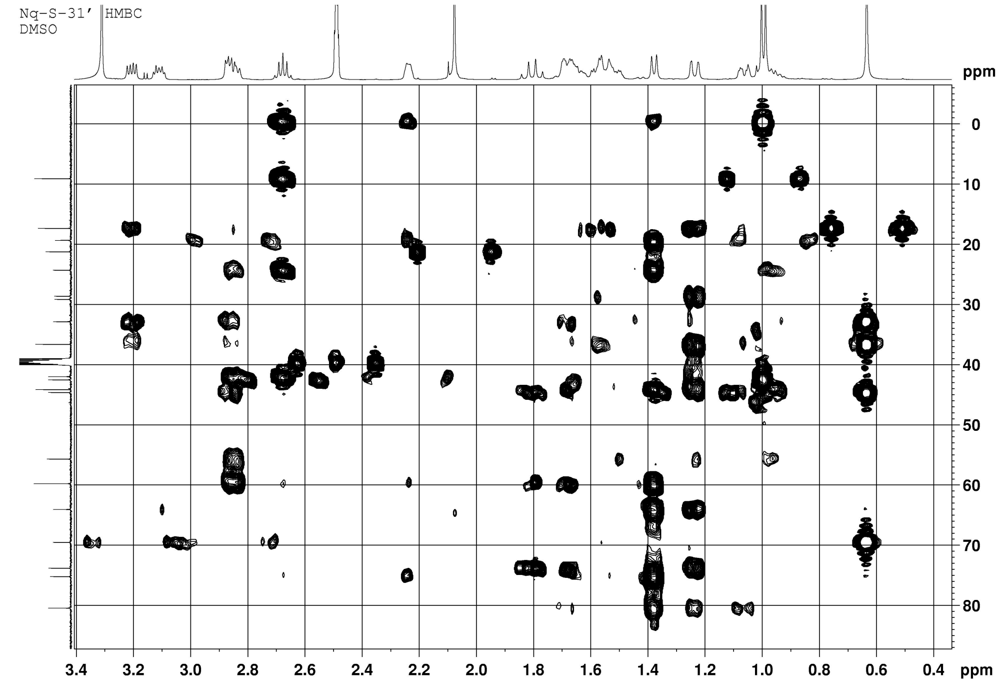

Figure S18. HMBC spectrum of compound **2**.

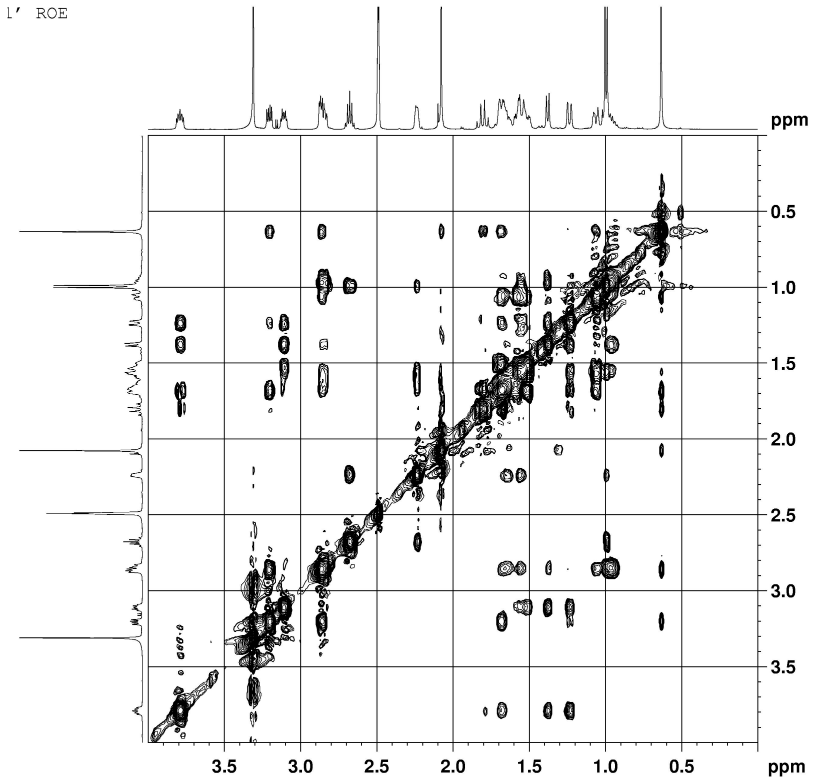

Figure S19. ROESY spectrum of compound **2**.

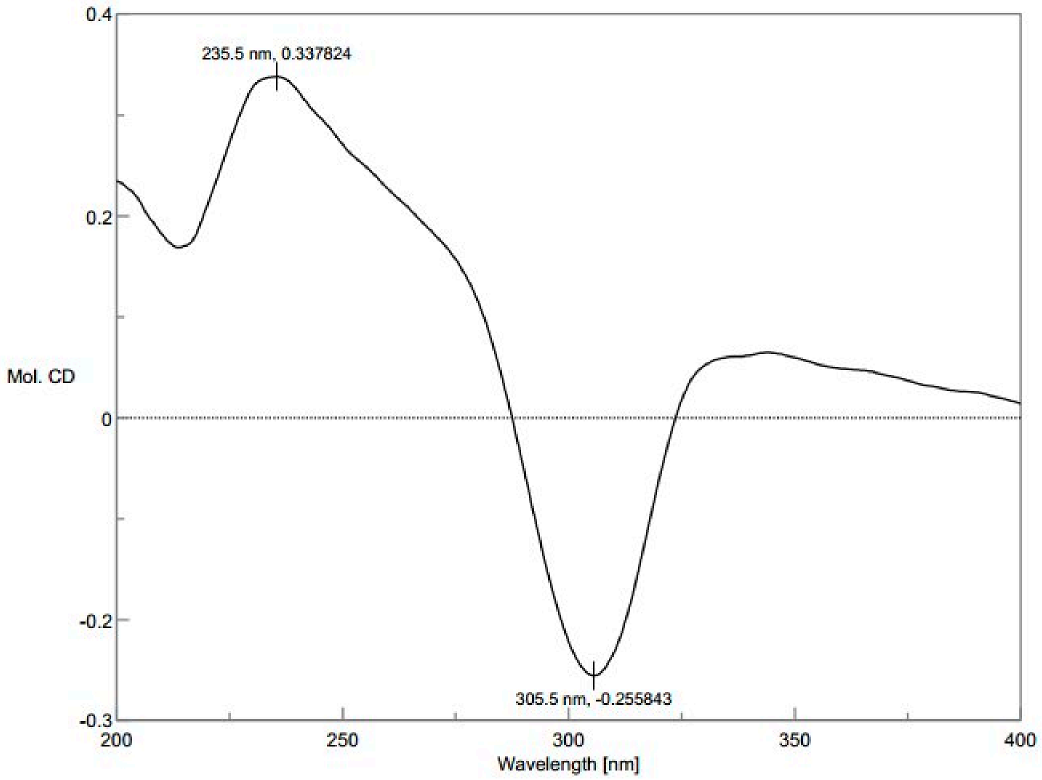

Figure S20. CD spectrum of compound **2**.

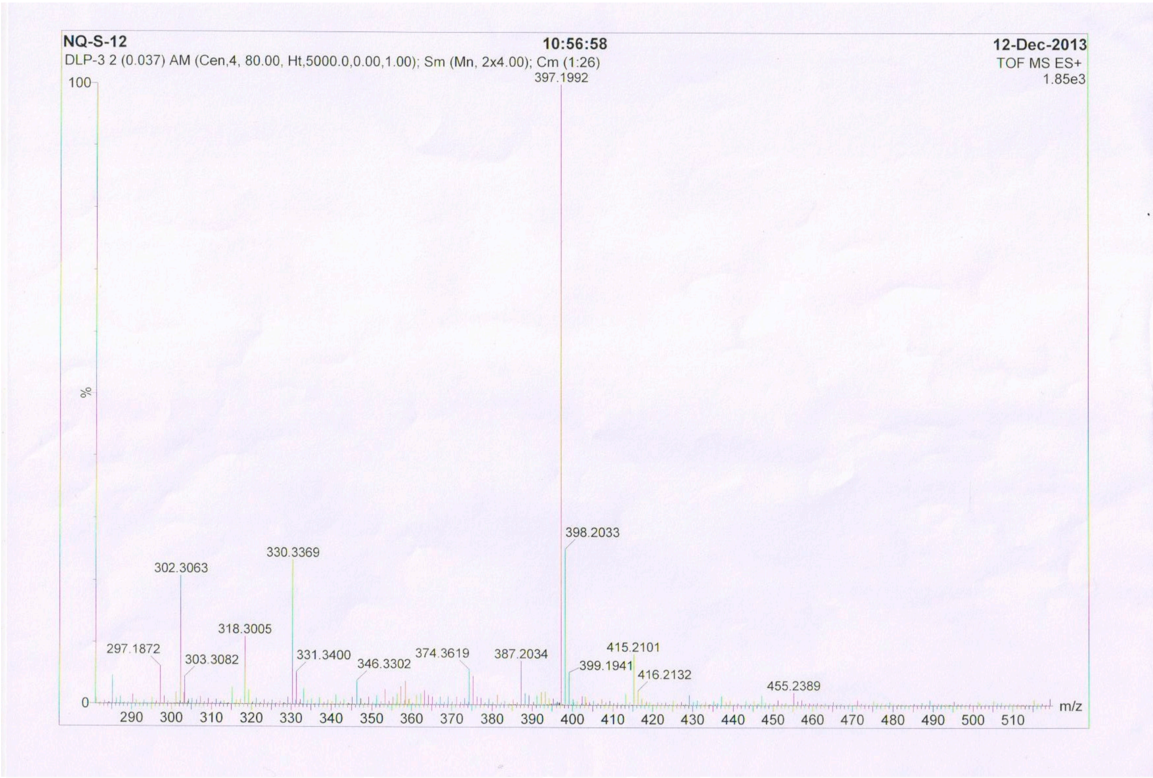

Figure S21. HR-ESI-MS spectrum of compound **3**.

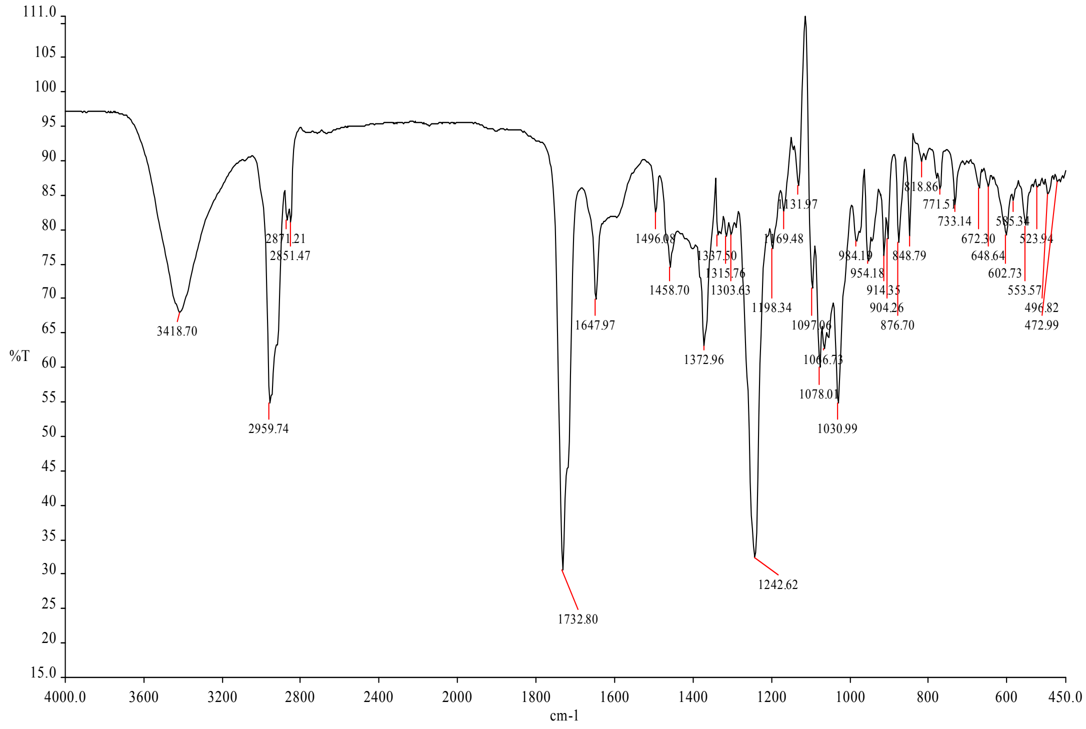

Figure S22. IR spectrum of compound **3**.

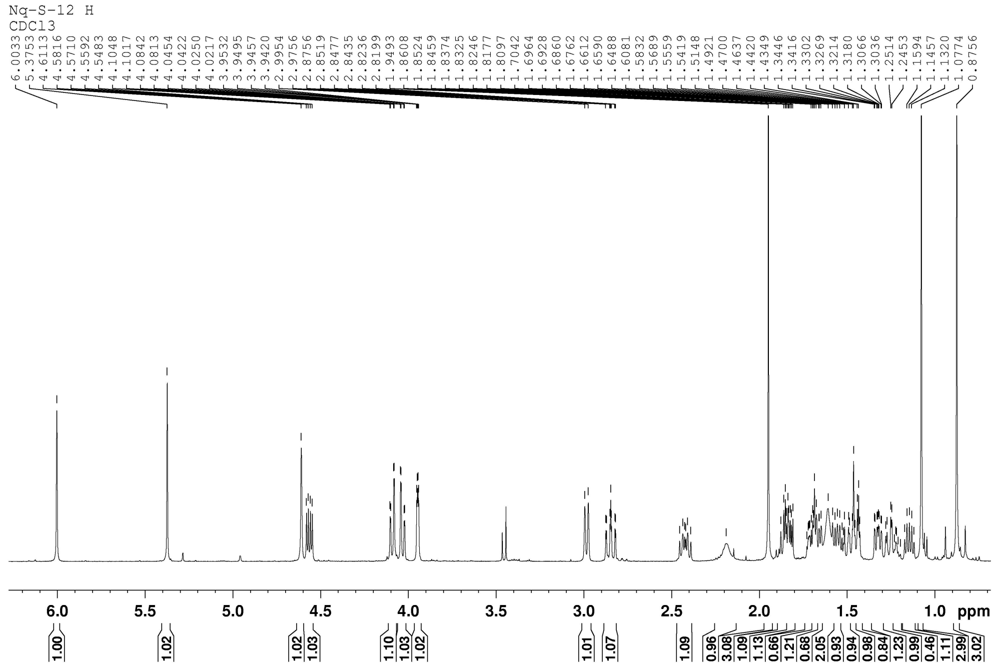

Figure S23. ^{1}H-NMR spectrum of compound **3**.

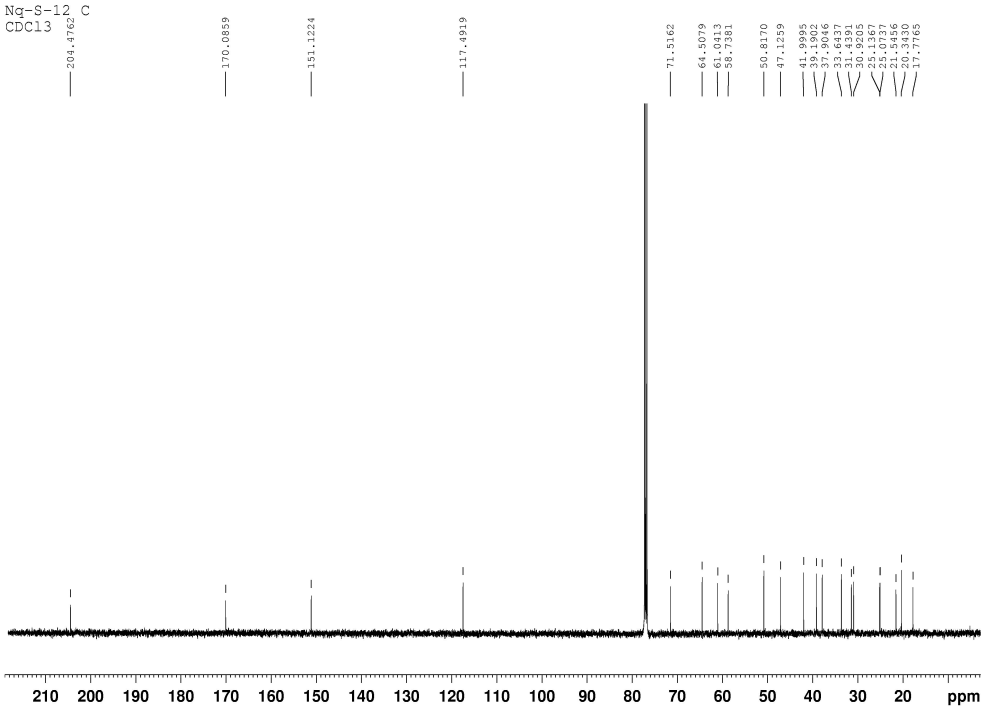

Figure S24. ^{13}C-NMR spectrum of compound **3**.

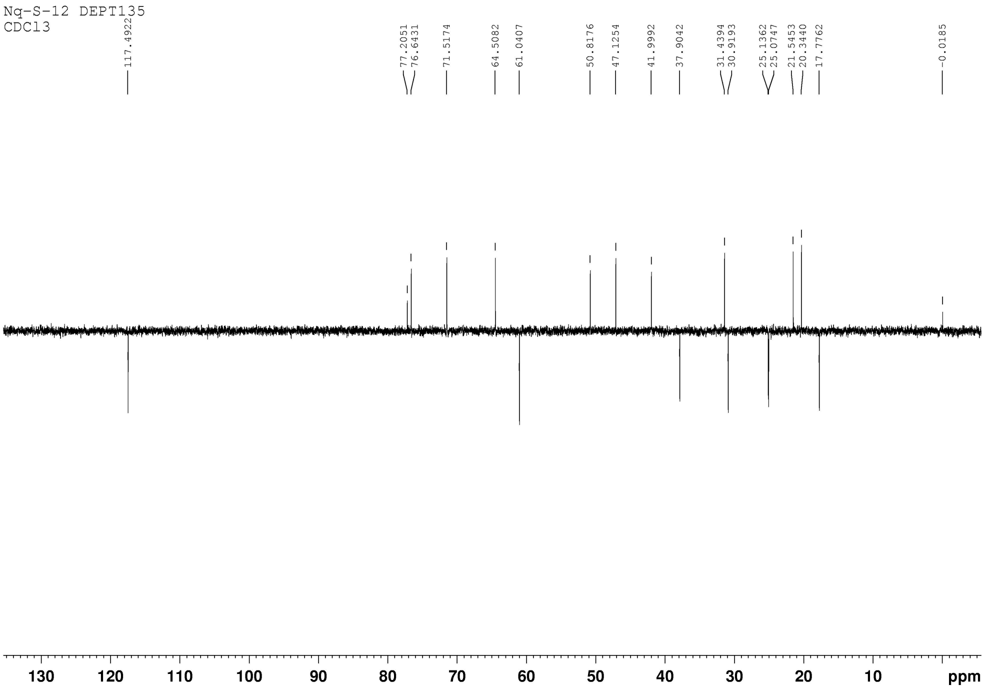

Figure S25. DEPT spectrum of compound **3**.

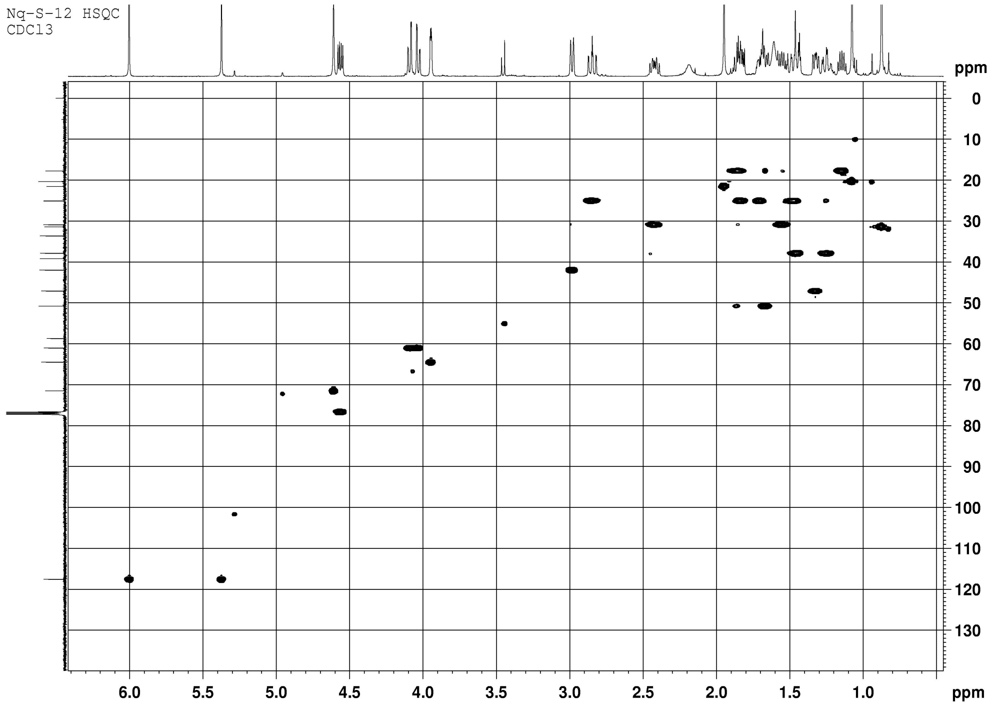

Figure S26. HSQC spectrum of compound **3**.

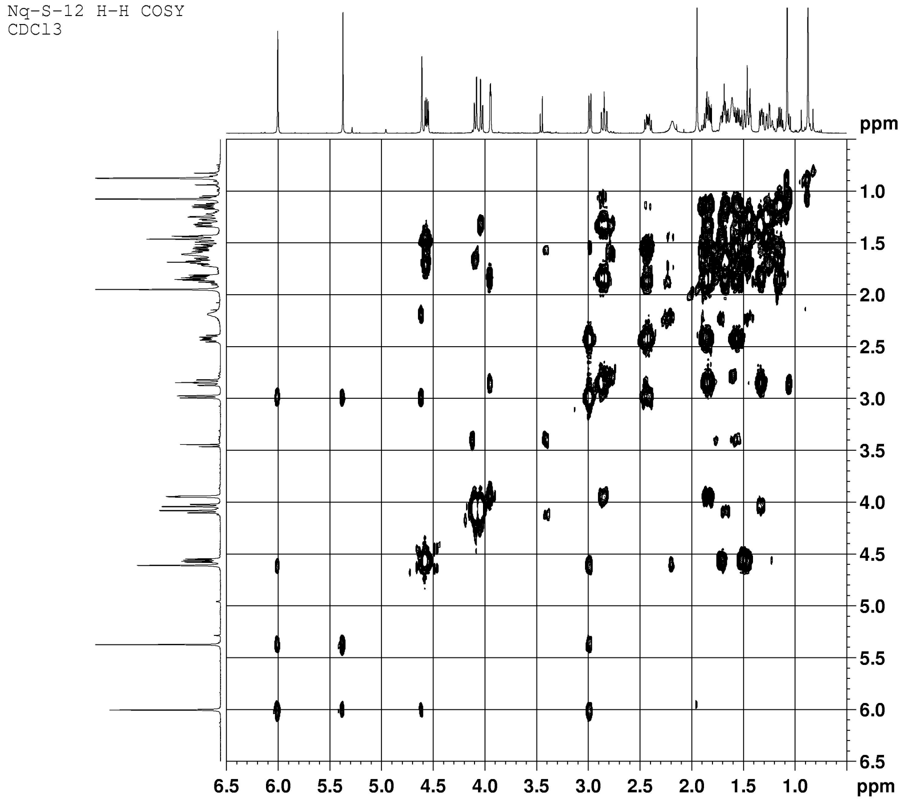

Figure S27. ^{1}H- ^{1}H COSY spectrum of compound **3**.

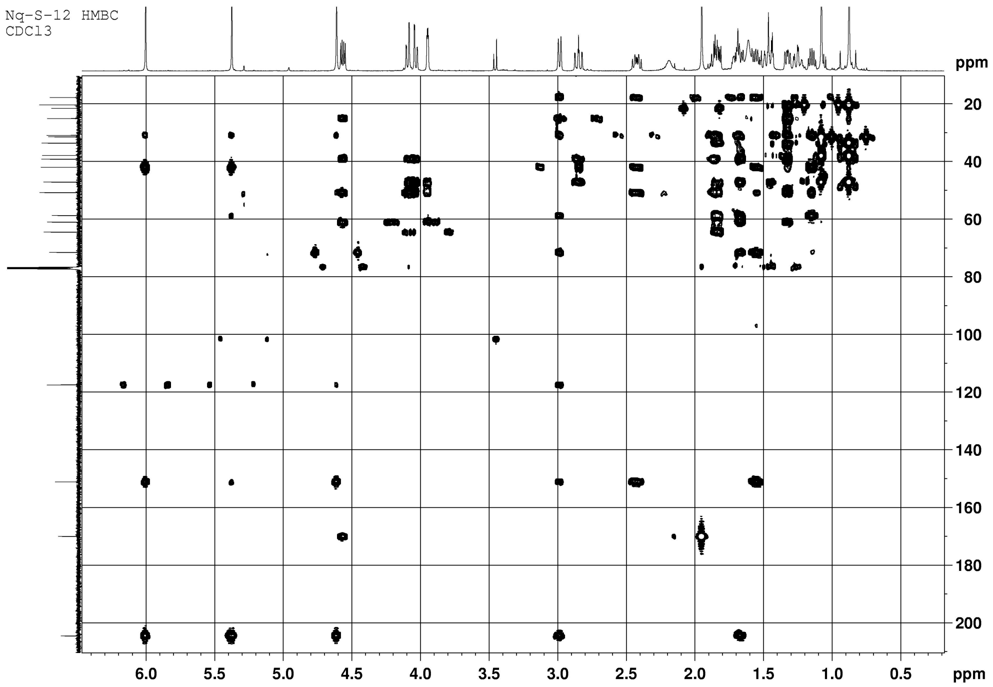

Figure S28. HMBC spectrum of compound **3**.

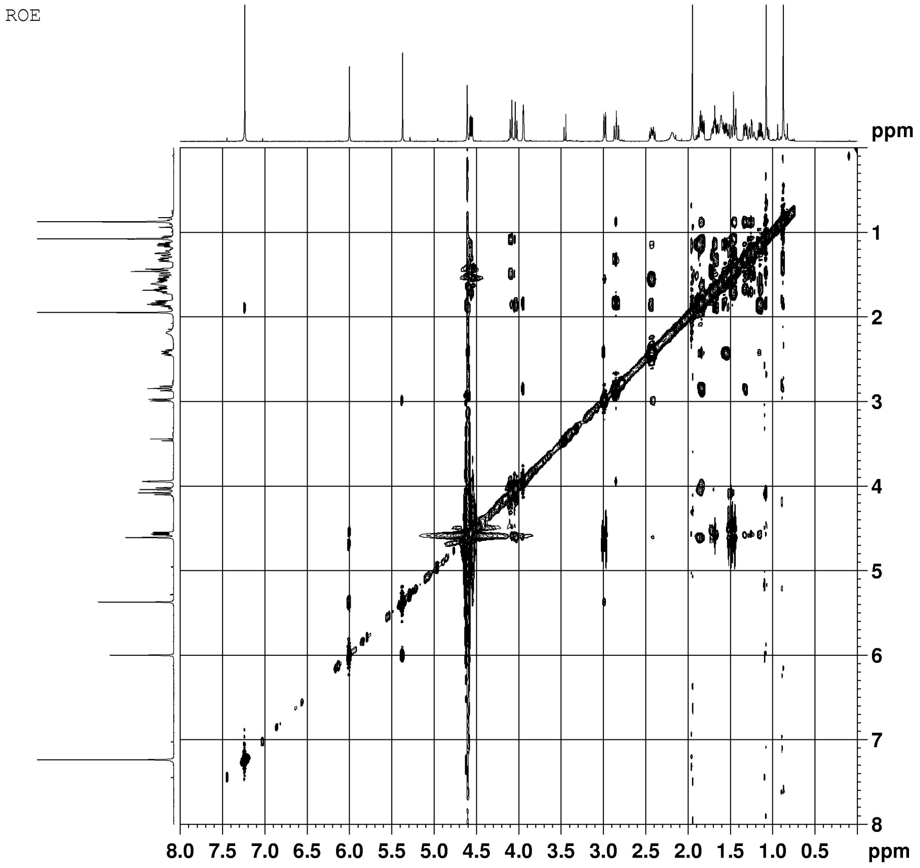

Figure S29. ROESY spectrum of compound **3**.

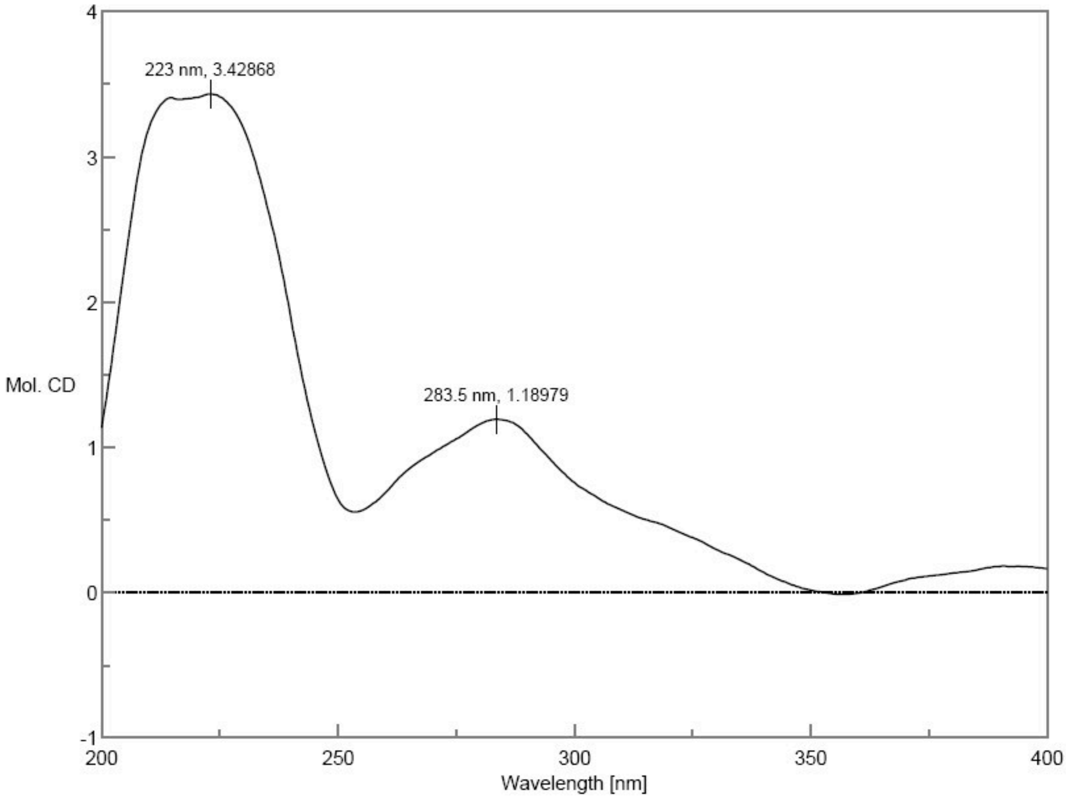

Figure S30. CD spectrum of compound **3**.
